# Complete human mtDNA genome sequences from Vietnam and the phylogeography of Mainland Southeast Asia

**DOI:** 10.1038/s41598-018-29989-0

**Published:** 2018-08-03

**Authors:** Nguyen Thuy Duong, Enrico Macholdt, Nguyen Dang Ton, Leonardo Arias, Roland Schröder, Nguyen Van Phong, Vo Thi Bich Thuy, Nguyen Hai Ha, Huynh Thi Thu Hue, Nguyen Thi Xuan, Kim Thi Phuong Oanh, Le Thi Thu Hien, Nguyen Huy Hoang, Brigitte Pakendorf, Mark Stoneking, Nong Van Hai

**Affiliations:** 1 Institute of Genome Research, Vietnam Academy of Science and Technology, 18 Hoang Quoc Viet, Cau Giay, Hanoi Vietnam; 2 Department of Evolutionary Genetics, Max Planck Institute for Evolutionary Anthropology, Deutscher Platz 6, D04103 Leipzig, Germany; 3 Dynamique du Langage, UMR5596, CNRS & Université de Lyon, 69363 Lyon, Cedex 07 France

## Abstract

Vietnam is an important crossroads within Mainland Southeast Asia (MSEA) and a gateway to Island Southeast Asia, and as such exhibits high levels of ethnolinguistic diversity. However, comparatively few studies have been undertaken of the genetic diversity of Vietnamese populations. In order to gain comprehensive insights into MSEA mtDNA phylogeography, we sequenced 609 complete mtDNA genomes from individuals belonging to five language families (Austroasiatic, Tai-Kadai, Hmong-Mien, Sino-Tibetan and Austronesian) and analyzed them in comparison with sequences from other MSEA countries and Taiwan. Within Vietnam, we identified 399 haplotypes belonging to 135 haplogroups; among the five language families, the sequences from Austronesian groups differ the most from the other groups. Phylogenetic analysis revealed 111 novel Vietnamese mtDNA lineages. Bayesian estimates of coalescence times and associated 95% HPD for these show a peak of mtDNA diversification around 2.5–3 kya, which coincides with the Dong Son culture, and thus may be associated with the agriculturally-driven expansion of this culture. Networks of major MSEA haplogroups emphasize the overall distinctiveness of sequences from Taiwan, in keeping with previous studies that suggested at most a minor impact of the Austronesian expansion from Taiwan on MSEA. We also see evidence for population expansions across MSEA geographic regions and language families.

## Introduction

Vietnam is part of Mainland Southeast Asia (MSEA), and is bordered by China to the north, Laos to the northwest, Cambodia to the southwest and the South China Sea to the east. The country is divided into three regions: Bac Bo (north); Trung Bo (central); and Nam Bo (south); and encompasses ~331,000 km^2^. According to the General Statistics Office of Vietnam, in 2017 the country was inhabited by ~93.7 million people (www.gso.gov.vn; accessed June 2017). Vietnam is ethnically diverse with 54 different recognized groups speaking languages belonging to five major language families [Austroasiatic (AA), Tai–Kadai (TK), Hmong–Mien (HM), Sino–Tibetan (ST), and Austronesian (AN)]. Most people speak AA languages (89.9% of the Vietnamese population); TK is the second most common (5.9%) followed by HM, ST and AN (2.1%, 1.2% and 0.9%, respectively)^1,2^. The AA family is widespread across the lowlands of Vietnam and MSEA, including Malaysia. The TK family is distributed throughout the northern highlands of Vietnam, Southern China, Myanmar, Cambodia, Thailand and Laos. The HM family has a scattered distribution over the northern highlands of Vietnam, southern China and Southeast Asia^1^, while the ST family is widespread across China and Myanmar but restricted to the northern highlands in Vietnam. In MSEA the AN family is mainly concentrated in highland and coastal areas of southern central Vietnam, but it is widespread across Taiwan and Island Southeast Asia^2^.

Geographically, MSEA consists of a number of very long river valleys, including the Chao Phraya, the Irrawaddy, the Mekong, the Red, and the Salween; most of these have their source in the eastern fringes of the Himalayas and follow a generally north-south direction^3^. River valleys facilitated the mobility of people and material goods throughout prehistory. The deltas of the Red River (located in northern Vietnam) and the Mekong (in southern Vietnam) lie only a few meters above sea level, are heavily populated, and largely agricultural^4^. In contrast, the northern and central regions of Vietnam are characterized by mountainous highlands, with peaks in excess of 3,000 meters, that are likely to have served as barriers to the movement of people.

The linguistic and geographic diversity found in Vietnam might have influenced the genetic diversity in this area, such as the mitochondrial DNA (mtDNA) variation in Vietnamese populations. However, most previous studies of Vietnamese mtDNA variation have sequenced only the hypervariable segments of the control region, and there are only a few complete mtDNA sequences available from Vietnamese populations^5–8^. New advances in next-generation sequencing have made it feasible to sequence and analyze large numbers of complete mtDNA genomes^9–11^, and we have used these methods to obtain complete mitochondrial genomes from 609 unrelated Vietnamese subjects that encompass all five language families (Figure S1, Online Resource 2). This is the first comprehensive study of complete mtDNA sequences of Vietnamese populations, with the goal of investigating the matrilineal ancestry of Vietnamese populations. Here we analyze the phylogeography of mtDNA haplogroups in MSEA, with an emphasis on the additional insights arising from this large sample of Vietnamese sequences; insights into the genetic history of specific Vietnamese populations will be described elsewhere.

## Methods

### Samples

A total of 609 blood samples from unrelated individuals speaking languages encompassing five language families (AA, TK, HM, ST, and AN; sample sizes in Table S1, Online Resource 1), were collected from Hanoi and the northeast and central highlands of Vietnam (Figure S1, Online Resource 2). All subjects self-identified as having at least three generations of the same ethnicity and all subjects gave written informed consent before blood collection. All experiments were performed in accordance with relevant guidelines and regulations based on the experimental protocol on human subjects which was approved by the Institutional Review Board of the Institute of Genome Research, Vietnam Academy of Science and Technology (No. 4-2015/NCHG-HDDD) and by the Ethics Commission of the University of Leipzig Medical Faculty.

### Mitochondrial DNA sequencing and multiple alignment

Genomic DNA was isolated from peripheral blood samples using a DNeasy Blood and Tissue Kit (Qiagen, Hilden, Germany) according to the manufacturer’s protocols. Double-indexed genomic libraries were constructed for each sample and capture-enrichment for mtDNA was carried out as described previously^12,13^. Sequencing was carried out on the Illumina platform and reads were processed and quality control measures carried out as described previously^14^. Reads were mapped to the Reconstructed Sapiens Reference sequence (RSRS)^15,16^ using an in-house alignment program and a multiple sequence alignment was performed using MAFFT^16^.

### Data analysis

Haplogroups were assigned using HaploGrep2^17^ with PhyloTree mtDNA tree Build 17^18^. Note that in the text haplogroup labels without an asterisk include all downstream subhaplogroups, whereas haplogroup labels with an asterisk exclude all downstream subhaplogroups. For instance, B4 refers to all haplotypes belonging to haplogroup B4, while B4* refers to haplotypes assigned to B4, but not assigned to any of the defined subhaplogroups within B4. A Bayesian skyline plot (BSP) and maximum clade credibility (MCC) trees were constructed using BEAST 1.8, based on Bayesian Markov chain Monte Carlo (MCMC). The software jModel test 2.1.7^19^ was used to select the best model for creation of the BEAST input file by BEAUTi v1.8^20^. The mtDNA genome was partitioned into the coding and noncoding regions with respective mutation rates of 1.708 × 10^−8^ and 9.883 × 10^−8^, and the RSRS sequence was used to root the mtDNA tree^21^. For constructing the BSP with Tracer we merged two BEAST runs (100 million steps each), using LogCombiner. A resampling of 30000 steps each for the log and trees file and a burn-in removal of 15 million steps were applied. The BSP was calculated for the 609 Vietnamese samples, excluding the RSRS, with the piecewise linear change parameter^22^. The Bayesian MCC trees from the BEAST runs were assembled with TreeAnnotator and drawn with FigTree v 1.4.0.

For comparison with the newly generated 609 mtDNA sequences from Vietnam, previously published data was assembled from Cambodia^23^, Laos^11^, Myanmar^24,25^, Malaysia^26^, Thailand^11^, Vietnam^6^, southern China^27^, and Taiwan^10^, as well as the data available at PhyloTree mtDNA tree Build 17^18^ (Table S1 in Online Resource 1). Hereafter we use “MSEA” to refer to this dataset. In total, 2742 complete mtDNA sequences were employed to construct the parsimony trees of complete mtDNA sequences by haplogroup, using the mtPhyl program (https://sites.google.com/site/mtphyl/). Median-joining networks of the major haplogroups were constructed with Network 5 (www.fluxus-engineering.com) and visualized in Network Publisher, and QGIS was used to construct contour maps depicting haplogroup frequencies (https://www.qgis.org/en/site/).

### Data availability

The sequences are available from GenBank (accession numbers: MH448947 - MH449555).

## Results and Discussion

### Vietnamese mtDNA haplogroups

We sequenced the entire mtDNA genome from 609 unrelated Vietnamese individuals to an average read depth of 840X (range: 80–5249). After alignment against the RSRS and haplotype assignment using Haplogrep2 and PhyloTree Build 17^4,18^, 399 distinct sequences (haplotypes) belonging to 135 haplogroups were identified, all belonging to the two macro-haplogroups M and N (Table S2 in Online Resource 1). Of the 135 haplogroups, 46 (33.82%) are singletons. Overall, F1 is the predominant haplogroup (19.38%) followed by B4 (17.41%), M7 (9.36%), B5 (7.22%), and M71 (6.08%); these haplogroups are also common in other MSEA populations^6,11,23,28^ (Table S3, S4 in Online Resource 1).

The distribution of major haplogroups with respect to language family is shown in Figure S2 (Online Resource 2) both for the Vietnamese populations alone and in combination with the MSEA dataset (including the present Vietnamese data). Since we provide the first complete mtDNA genome sequences for HM populations in MSEA, the HM haplogroup distributions are identical for Vietnam (Figure S2a, Online Resource 2) and MSEA (Figure S2b, Online Resource 2). For Vietnam, the most striking feature is the difference between AN groups and the other four language families: A and B4 are lacking in AN groups but comprise 1.12–33.58% of the haplogroups in the other language families, while M71 is at much higher frequency in AN groups (37.04%) than in the other language families (0–19.32%) (Table S3 in Online Resource 1). The ST groups stand out in having a higher frequency of B4 (33.58%) than do AA, TK, or HM groups (7.95–18.99%), while the HM groups have relatively high frequencies of haplogroups A (7.14%) and G (5.84%). The AN groups from Vietnam (Figure S2a, Online Resource 2) also show a different haplogroup composition than the MSEA AN groups (Figure S2b, Online Resource 2); the Vietnamese AN groups have a much higher frequency of M71 and lack B4, E and F4, which have frequencies of 16.67%, 8.83% and 4.33% respectively in the MSEA AN groups (Table S4 in Online Resource 1), with Taiwan contributing most of these (Table S1 in Online Resource 1). Haplogroups E and B4 are also important haplogroups in Island Southeast Asia^29–33^: haplogroup E has been reported at frequencies of 9.7–20.34% in the Philippines^29–31^ and 2.2–26.5% across Indonesia^29,30^ while B4 has been reported at frequencies of 12.9–35.58% in the Philippines^29–31^ and 2.0–28% across Indonesia^29,30^. Among the language families in MSEA, the AA groups are the most distinctive, as they have the lowest frequency of B4 and (along with AN groups) a relatively high frequency of M71 (Figure S2b, Online Resource 2).

### Novel Vietnamese mtDNA lineages

To identify Vietnamese-specific lineages (clades or branches consisting of sequences only from Vietnam), we constructed phylogenetic trees relating 2742 entire mtDNA genome sequences (including 609 newly sequenced mitogenomes from the present study and 2133 previously reported sequences from MSEA). Several previously undescribed sub-branches of haplogroups A, B, C, D, F, M and N were identified (Figures S5–S16 in Online Resource 2). In total, 111 novel lineages unique to Vietnam were found in the dataset. The majority of these belong to haplogroups B, F and M (25, 26 and 29, respectively); these are major haplogroups of MSEA, accounting for 76.35% of the sequences. A total of 41 of the unique Vietnam lineages contain internal branching events (i.e. comprise two or more haplotypes); these were used to estimate the coalescence times and associated 95% HPD for each such Vietnam-specific lineage (Table S5 in Online Resource 1). The distribution of coalescence times for Vietnam-specific lineages shows a peak around 2.5–3 kya (Figure S3, Online Resource 2). A Bayesian skyline plot (BSP; Figure S4, Online Resource 2) of population size change through time, based on all of the Vietnamese sequences, shows a peak of population expansion at around the same time. Given the uncertainty associated with both the population expansion time in the BSP (Figure S4, Online Resource 2) and the coalescence time estimates (Table S5, Online Resource 1), these dates are broadly consistent with each other. Archaeological evidence indicates that the Dong Son culture expanded throughout Vietnam beginning about 2.6 kya^34^, in association with rice agriculture, and so the patterns we see in mtDNA diversification and expansion might be related to this cultural expansion. The apparent decline in population size in the BSP beginning around 3–4 kya is probably an artifact due to sampling and population substructure, as has been shown in previous studies^35^.

### Coalescence times of MSEA mtDNA haplogroups

We estimated coalescence ages of the MSEA mtDNA haplogroups and their subhaplogroups by combining all available data from Thailand, Laos, Cambodia, Myanmar, west Malaysia, and southern China, as well as Taiwan (Table S1 in Online Resource 1). The coalescence times and 95% HPD intervals are shown in Table S6 (Online Resource 1). The coalescence ages of the major haplogroups of MSEA, namely B, F, and M, are about ~49 kya, ~50 kya, and ~58 kya, respectively, consistent with the suggested presence of modern humans in Southeast Asia by 51–46 kya^36^. Moreover, the addition of our large sample of Vietnamese mtDNA sequences has resulted in deeper ages for several haplogroups that were poorly sampled in previous studies. For example, we estimate that haplogroup B5 coalesces at ~42 kya in MSEA (Table S6, Online Resource 1), compared to previous estimates of ~34 kya^23^ and ~36 kya^11^. Haplogroups M21 and M74 both coalesce at 44 kya in MSEA, much older than previous estimates of ~26 kya^23^ and ~34 kya^11^. M68 coalesces at 29 kya, compared to previous estimates of ~16 kya^11^ and ~20 kya^23^.

### Phylogeographic patterns of the MSEA mtDNA haplogroups

One of the most significant contributions of this study is to add 609 complete mtDNA sequences from Vietnam to previously generated data from other parts of MSEA. To explore additional insights into the phylogeographic patterns of the major MSEA haplogroups in relation to geography and language family, contour maps and networks were constructed for each major haplogroup and are discussed below.

### The phylogeography of haplogroup A

**Figure 1:**
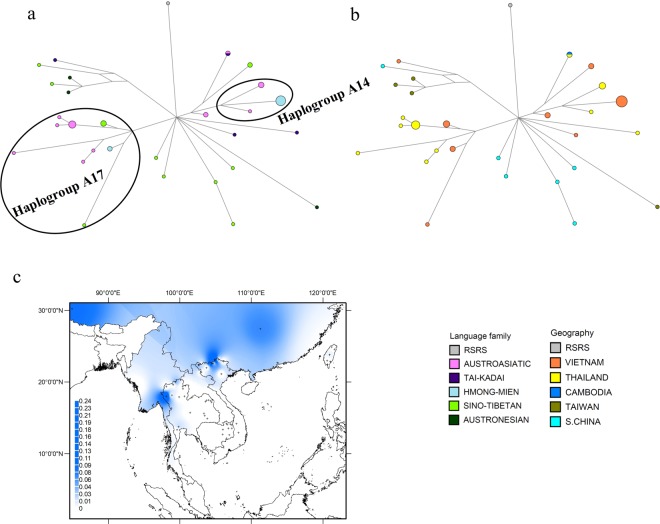
Diversity and distribution of haplogroup A. (**a**,**b**) Network of haplogroup A complete mtDNA sequences, color-coded by linguistic affiliation and geographic origin, respectively. (**c**) Spatial frequency distribution map of haplogroup A in MSEA: the more intense the color, the higher the frequency in the population. Small crosses mark the locations of the 68 MSEA populations included in the analysis (see Table S1 in Online Resource 1).

Haplogroup A occurs mostly in northern and eastern Asia at frequencies from 5 to 10%^37^, and is one of five founder haplogroups among native Americans^38^. Of our 21 new haplogroup A sequences, 15 belong to subhaplogroups A14 and A17, and define three new sub-branches (branches 1–3 in Figure S5, Online Resource 2), one of which (branch 2) shows internal divergence. In MSEA, A14 and A17 coalesce ~9 kya and ~14 kya, respectively (Table S6, Online Resource 1). Overall, haplogroup A is most widespread in AA groups from Vietnam and Thailand, with additional haplotypes in AN groups from Taiwan (Fig. 1a,b). Within MSEA, haplogroup A is at highest frequency in northern Vietnam and northwestern Thailand (Fig. 1c).

### The phylogeography of haplogroup B

**Figure 2:**
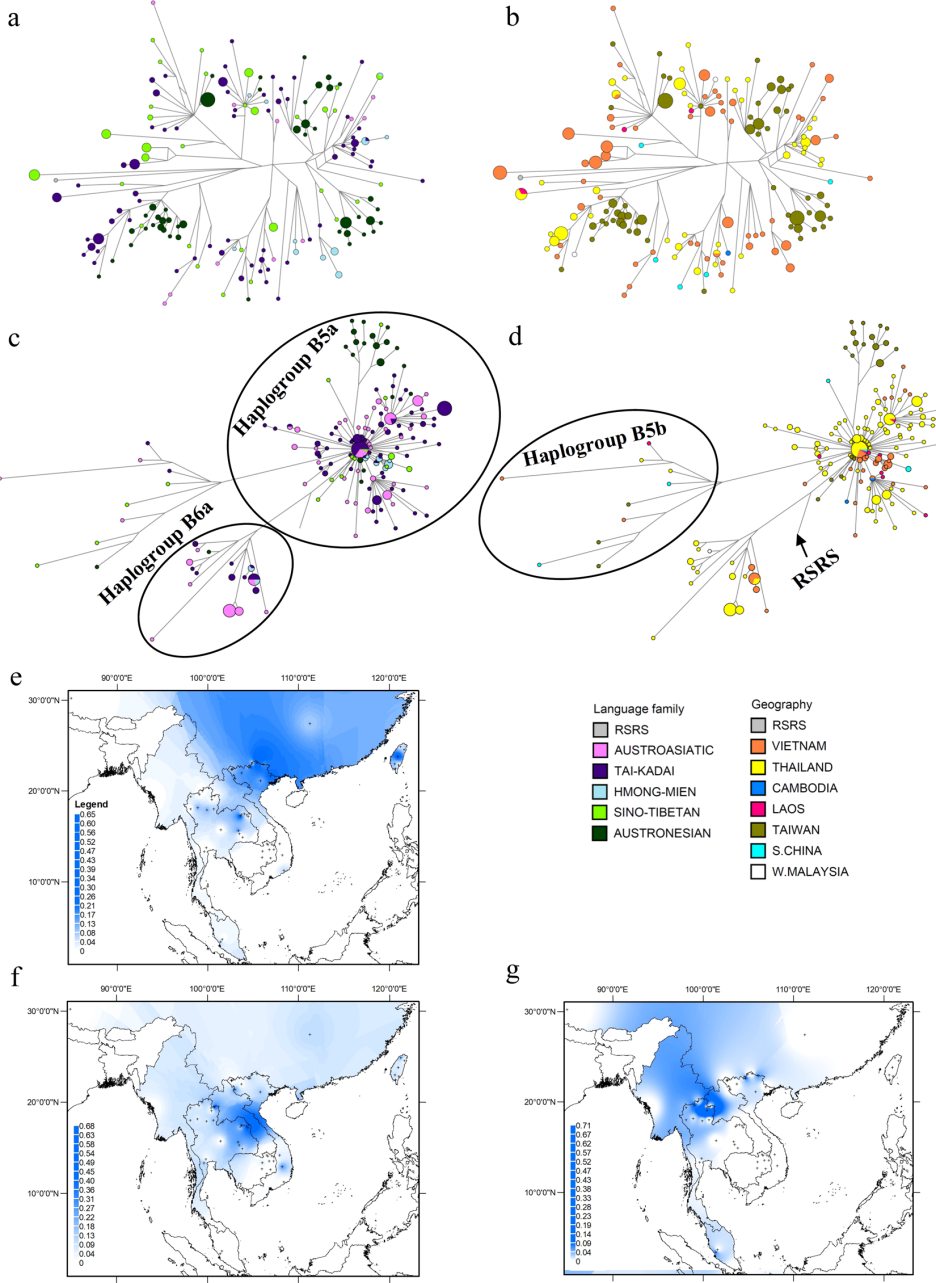
Diversity and distribution of subhaplogroups B4, B5, and B6a. (**a**,**b**) Network of haplogroup B4 complete mtDNA sequences. (**c**,**d**) Network of haplogroup B5 and B6a complete mtDNA sequences, color-coded by linguistic affiliation and geographic origin, respectively. (**e**–**g**) Spatial frequency distribution maps of haplogroups B4, B5 and B6a in MSEA, respectively: the more intense the color, the higher the frequency in the population. Crosses mark the sampling sites of the 68 MSEA populations included in the analysis (see Table S1 in Online Resource 1).

Haplogroup B is one of the most common haplogroups in northern and eastern Asia^37^, with three major subhaplogroups B4, B5 and B6^18^. In MSEA, B coalesces at ~49 kya, B4 at ~40 kya, B5 at ~42 kya, and B6a - the only subhaplogroup of B6 found in this region - at ~23.5 kya (Table S6, Online resource 1). With the 164 Vietnamese mtDNA sequences belonging to haplogroup B, several new sub-clusters within B4, B5, and B6a are identified (Figures S6 and S7 in Online Resource 2). B4 is the second most frequent haplogroup in Vietnam and is widespread across MSEA, especially northern Vietnam, northern Thailand, and Taiwan (Fig. 2e)^6,11,23,28^. However, B4 subhaplogroups that are relatively frequent in Taiwan (e.g. B4a1a, B4a2a1, B4a2a3, B4b1a2g, B4b1a2g1, B4c1b2a2a)^10^ are absent in the Vietnamese AN groups (Table S1 in Online Resource 1). Moreover, subhaplogroups B4*, B4b1c1*, B4c*, B4c1a*, B4g1* are only present in Vietnamese populations (marked with numbers 10, 3, 5, 4, and 9, respectively, in Figure S6, Online Resource 2) but are absent in the Vietnamese AN populations. Thus, while haplogroup B4 has the highest frequency in northern Vietnam and Taiwan (Fig. 2e), there is very little overlap of B4 subhaplogroups between Vietnam and Taiwan (Table S1 in Online Resource 1). Overall, there is remarkably little sharing of sequences between groups from different language families or countries; one haplotype is shared between Vietnamese TK and HM groups and another is shared between Vietnamese ST and HM groups, while two haplotypes are shared between Vietnam and Thailand and one between Thailand and Laos, all from TK-speaking groups (Fig. 2a,b). These results suggest a relatively old spread of haplogroup B4 across MSEA.

The phylogeny of haplogroups B5 and B6a is shown in Figure S7 (Online Resource 2); major subhaplogroups in MSEA are B5a1a, B5a1c1a1 and B6a^10,11,27^. In contrast to haplogroup B4, the network for subhaplogroup B5a shows a star-like pattern with sharing of identical or closely-related sequences across different language families and geographic regions (Fig. 2c,d), reflecting a likely population expansion in MSEA^6,11,23,28^. There is also a distinct cluster of haplotypes from Taiwan in subhaplogroup B5a. B5b shows a different pattern with no sharing of diverse sequences found in individuals from a variety of countries and speaking languages belonging to different families. Overall, haplogroup B5 reaches the highest frequency in northeastern Thailand (Fig. 2f).

Haplogroup B6a, found in 14 Vietnamese and 26 Thai samples, comprises four sister sub-branches (Figure S7, Online Resource 2). This haplogroup occurs mostly in AA and TK groups from Thailand and also is found in an AN individual from West Malaysia; a divergent haplotype is found in an AA individual from Thailand (Fig. 2c,d)^11,26^. In addition, a haplotype is shared between AA, TK, and HM groups from Vietnam and Thailand. Haplogroup B6a is distributed mostly in northern Thailand (Fig. 2g).

### The phylogeography of haplogroup C

**Figure 3:**
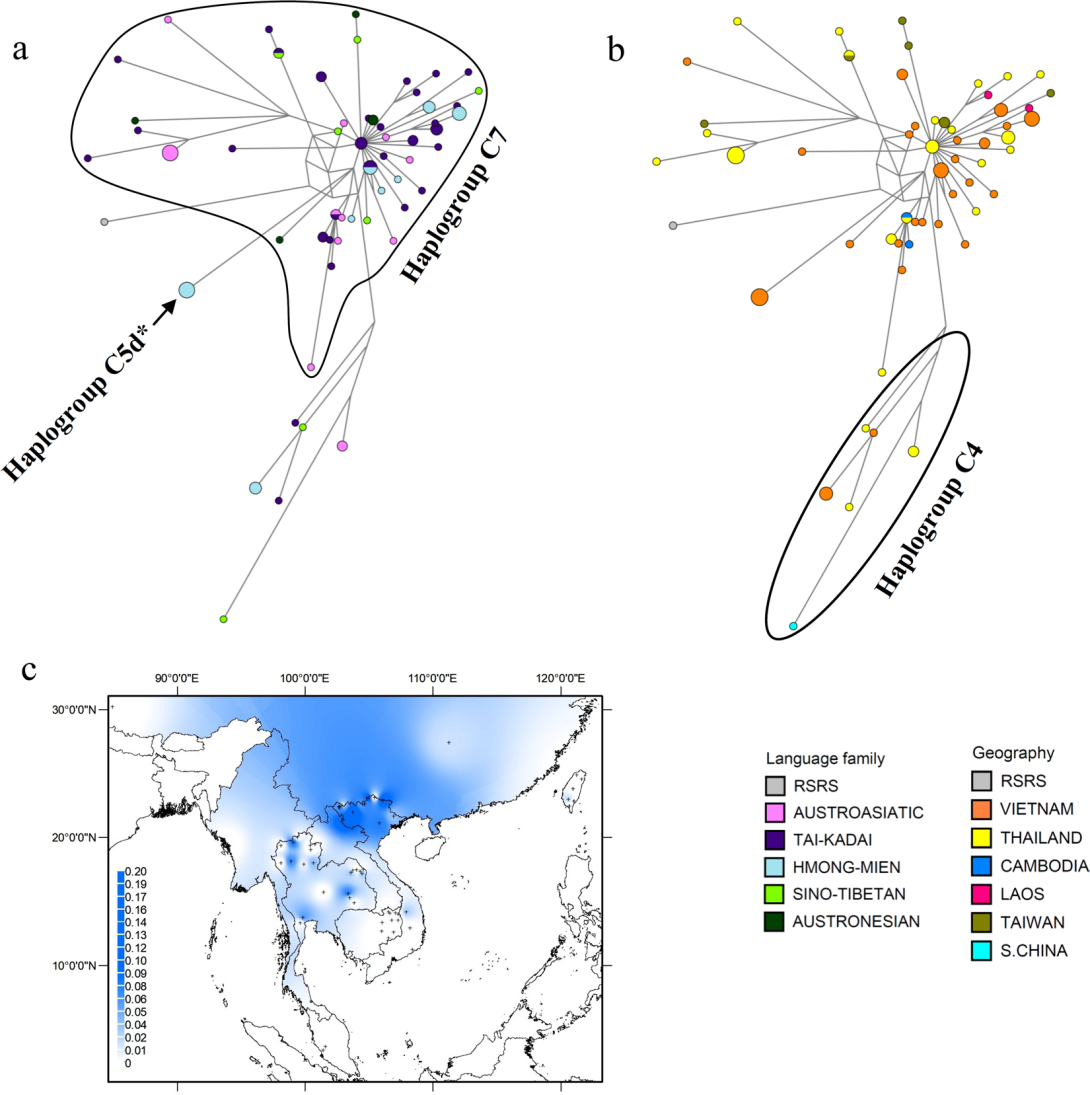
Diversity and distribution of haplogroup C. (**a**,**b**) Network of haplogroup C complete mtDNA sequences, color-coded by linguistic affiliation and geographic origin, respectively. (**c**) Spatial frequency distribution map of haplogroup C in MSEA: the more intense the color, the higher the frequency in the population. Crosses mark the sampling sites of the 68 MSEA populations included in the analysis (see Table S1 in Online Resource 1).

Haplogroup C is widespread across East Asia and is one of the five founder haplogroups among native American populations^38,39^; major subhaplogroups in MSEA are C4, C5d, and C7 (Figure S8, Online Resource 2). In this region, haplogroup C has a coalescence time estimate of ~23 kya, C4 of 15.9 kya, C5d of 2.7 kya and C7 of ~17 kya (Table S6 in Online Resource 1). The 41 newly generated haplogroup C sequences identify several novel lineages belonging to haplogroup C7a (branches 2–12, Figure S8 in Online Resource 2), of which five have internal divergences and were hence used for coalescence time estimates (branches 3, 5, 8, 9 and 12; Figure S8 in Online Resource 2). There is also one new lineage belonging to C4a2b* (branch 1, Figure S8 in Online Resource 2) and a novel lineage C5d* (Table S5 in Online Resource 1; Figure S8 in Online Resource 2). Haplogroup C reaches the highest frequency in northwestern Vietnam (Fig. 3c). Most (~84%) of the sequences belong to C7 and network analysis shows a star-like pattern, suggesting expansion (Fig. 3a,b). This haplogroup has a patchy distribution in AN groups from Taiwan and in Vietnamese individuals from all five language families. Three haplotypes are each shared between TK individuals and individuals from one other language family (AA, HM, and ST, respectively). Haplogroup C5 is represented by a single haplotype belonging to subhaplogroup C5d* (branch 17 in Figure S8, Online Resource 2) and is present only in Vietnamese HM groups, while C4 is distributed in Vietnamese, Thai, and S. Chinese groups from all language families except AN (Fig. 3a,b).

### The phylogeography of haplogroup D

**Figure 4:**
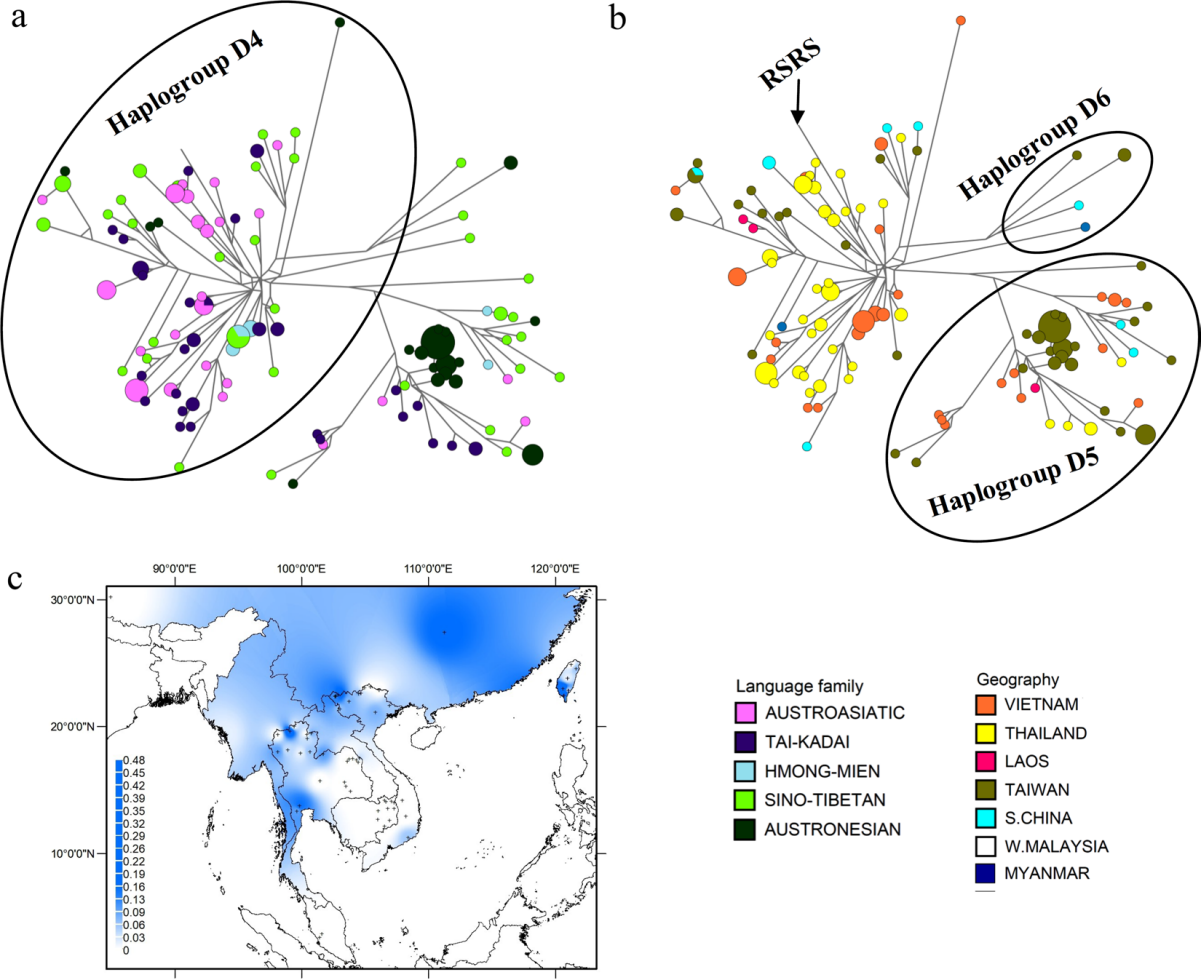
Diversity and distribution of haplogroup D. (**a**,**b**) Network of haplogroup D complete mtDNA sequences, color-coded by linguistic affiliation and geographic origin, respectively. (**c**) Spatial frequency distribution map of haplogroup D in MSEA: the more intense the color, the higher the frequency in the population. Crosses mark the sampling sites of the 68 MSEA populations included in the analysis (see Table S1 in Online Resource 1).

Haplogroup D is found in Northeast Asia and Central Asia^40,41^ and is also one of the founding haplogroups of the New World^42,43^; in MSEA it coalesces at ~38.5 kya (Table S6 in Online Resource 1). Phylogenetic analysis reveals four new sub-branches (labelled 1–4 in Figure S9, Online Resource 2). Of 174 sequences from haplogroup D in the MSEA dataset, 59% belong to D4. This haplogroup is distributed in groups from all five language families and network analysis shows a signature of an expansion, with two haplotypes shared by groups from different language families: one shared between AA and TK groups from Thailand, and the other between HM and ST groups from Vietnam (Fig. 4a,b). D5 is also found in groups from all five language families, with the highest frequency in AN speakers from Taiwan (Fig. 4a,b). Haplogroup D reaches the highest frequency in southern Taiwan (Fig. 4c).

### Phylogeography of haplogroup F

**Figure 5:**
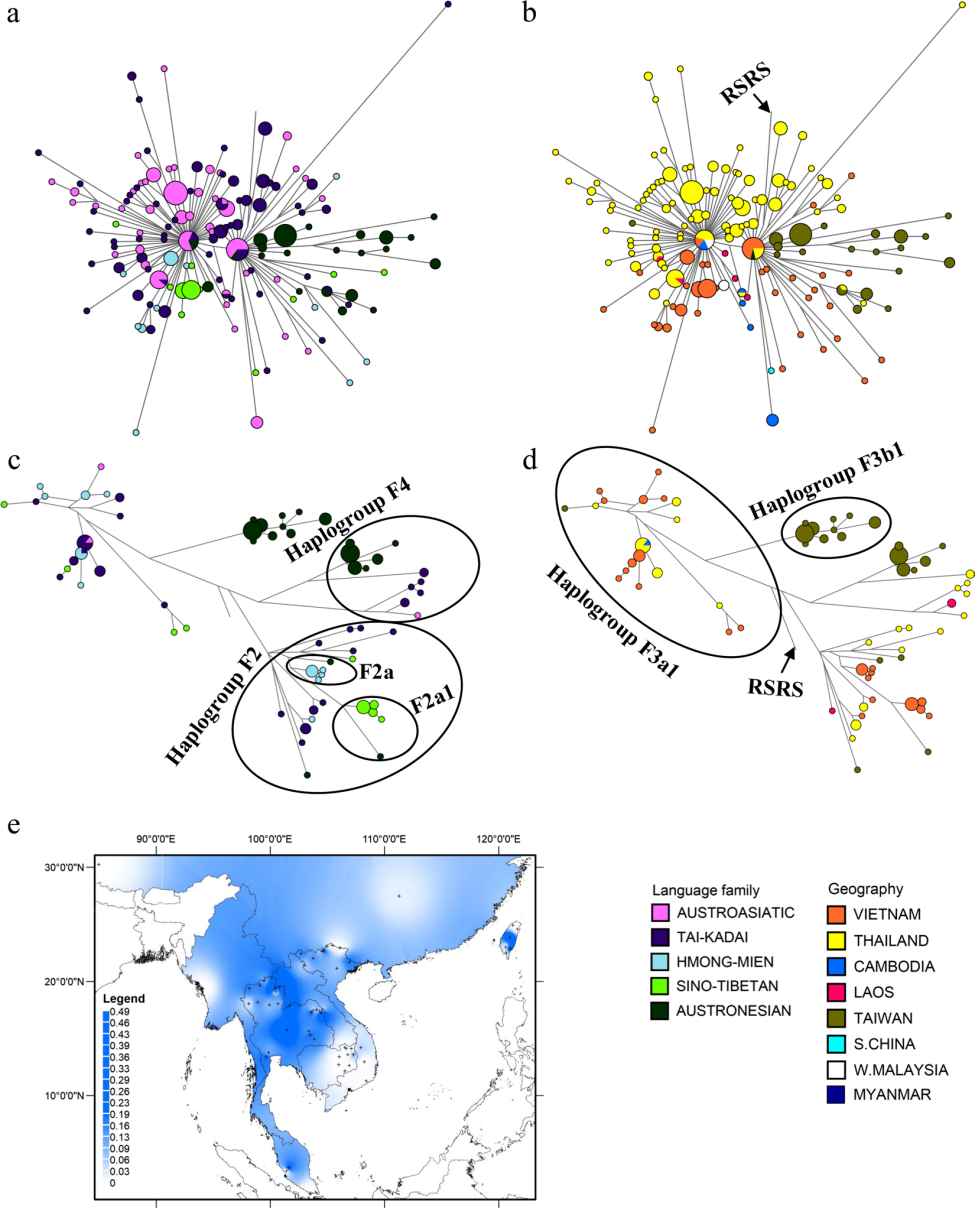
Diversity and distribution of the subhaplogroups of haplogroup F found in MSEA. (**a**,**b**) Network of haplogroup F1a complete mtDNA sequences color-coded by linguistic affiliation and geographic origin, respectively. (**c**,**d**) Network of haplogroup F2–4 complete mtDNA sequences, color-coded by linguistic affiliation and geographic origin, respectively. (**e**) Spatial frequency distribution map of haplogroup F1a in MSEA: the more intense the color, the higher the frequency in the population. Crosses mark the sampling sites of the 68 MSEA populations included in the analysis (see Table S1 in Online Resource 1).

Haplogroup F is one of the most common haplogroups throughout Asia, with frequencies ranging from 31 to 77%^6,29,41,44^ and a coalescence time of ~50 kya (Table S6 in Online Resource 1). The major subhaplogroups in our data are F1(xF1a1a), F1a1a, F2, and F3a1 (Table S2; Table S3 in Online Resource 1; Figures S10–S12 in Online Resource 2). The 154 Vietnamese sequences belonging to haplogroup F identify 26 new lineages, of which 12 show internal divergences and were used for coalescence time estimates (Table S5 in Online Resource 1). Major subhaplogroups in MSEA are F1(xF1a), F1a, F2, F3 and F4 (Table S4, Online Resource 1). The F1a sequences coalesce at ~18 kya (Table S6 in Online Resource 1), and network analysis shows a star-like pattern with numerous shared sequences, indicative of a recent expansion (Fig. 5a,b). Haplogroup F1a is at high frequency in northern Vietnam and northeastern Thailand (Fig. 5e).

The other major branches (F2, F3 and F4) have coalescence times in MSEA of ~24 kya, ~34 kya and ~38 kya, respectively. Subhaplogroups F2a and F2a1 include one AN sequence each from Taiwan, but are otherwise restricted to Vietnamese HM and ST groups, respectively (Fig. 5c,d). Haplogroup F3 is found in groups from all five language families; in AN groups from Taiwan it is represented solely by subhaplogroup F3b1. One haplotype of F3a1 is shared between TK and HM Vietnamese groups, and another is shared between Cambodian AA and Thailand TK groups. Haplogroup F4 is most frequent in AN groups from Taiwan and also occurs in a few TK and AA individuals from Thailand and Laos (Fig. 5c,d).

### Phylogeography of haplogroup G

**Figure 6:**
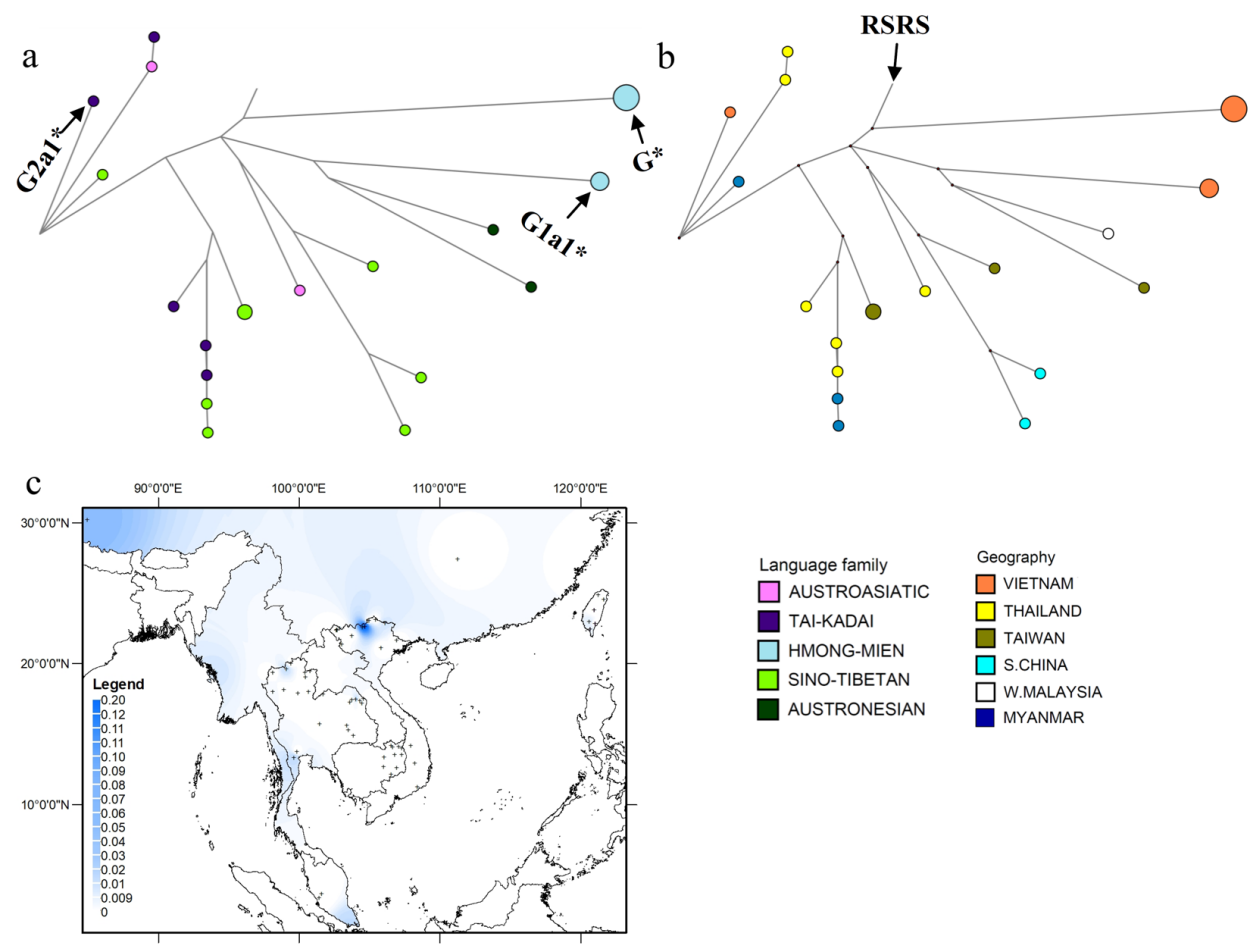
Diversity and distribution of haplogroup G. (**a**,**b**) Network of haplogroup G complete mtDNA sequences, color-coded by linguistic affiliation and geographic origin, respectively. (**c**) Spatial frequency distribution map of haplogroup G in MSEA: the more intense the color, the higher the frequency in the population. Crosses mark the sampling sites of the 68 MSEA populations included in the analysis (see Table S1 in Online Resource 1).

Haplogroup G is one of the most common mtDNA haplogroups among Japanese, Ainu, Mongol and Tibetan populations, and is also found at a lower frequency across East Asia, Central Asia and MSEA^11,45–47^. Haplogroup G in MSEA has a coalescence time of ~29 kya (Table S6 in Online Resource 1). The 10 Vietnamese haplogroup G sequences represent three new lineages: three identical sequences represent a new sub-branch of G1a1*, one represents a new sub-branch of G2a1*, and six identical sequences define a new lineage of G*, characterized by 13 shared mutations (these are labeled as branches 1–3 respectively in Figure S13 in Online Resource 2). The G2a1* sequence is from a TK group, and the other new lineages are restricted to HM groups (Fig. 6a,b). Among MSEA populations, haplogroup G reaches the highest frequency in northern Vietnam (Fig. 6c).

### Phylogeography of haplogroup M

M is a macro-haplogroup found at high frequency all across Asia (including MSEA)^11,48–50^; the coalescence time is ~58 kya (Table S6 in Online Resource 1). The 147 Vietnamese haplogroup M sequences fall into 38 subhaplogroups, and 63.95% of the sequences belong to M7 and M71 (Tables S2 and S3 in Online Resource 1; Figures S14 and S15 in Online Resource 2).

**Figure 7:**
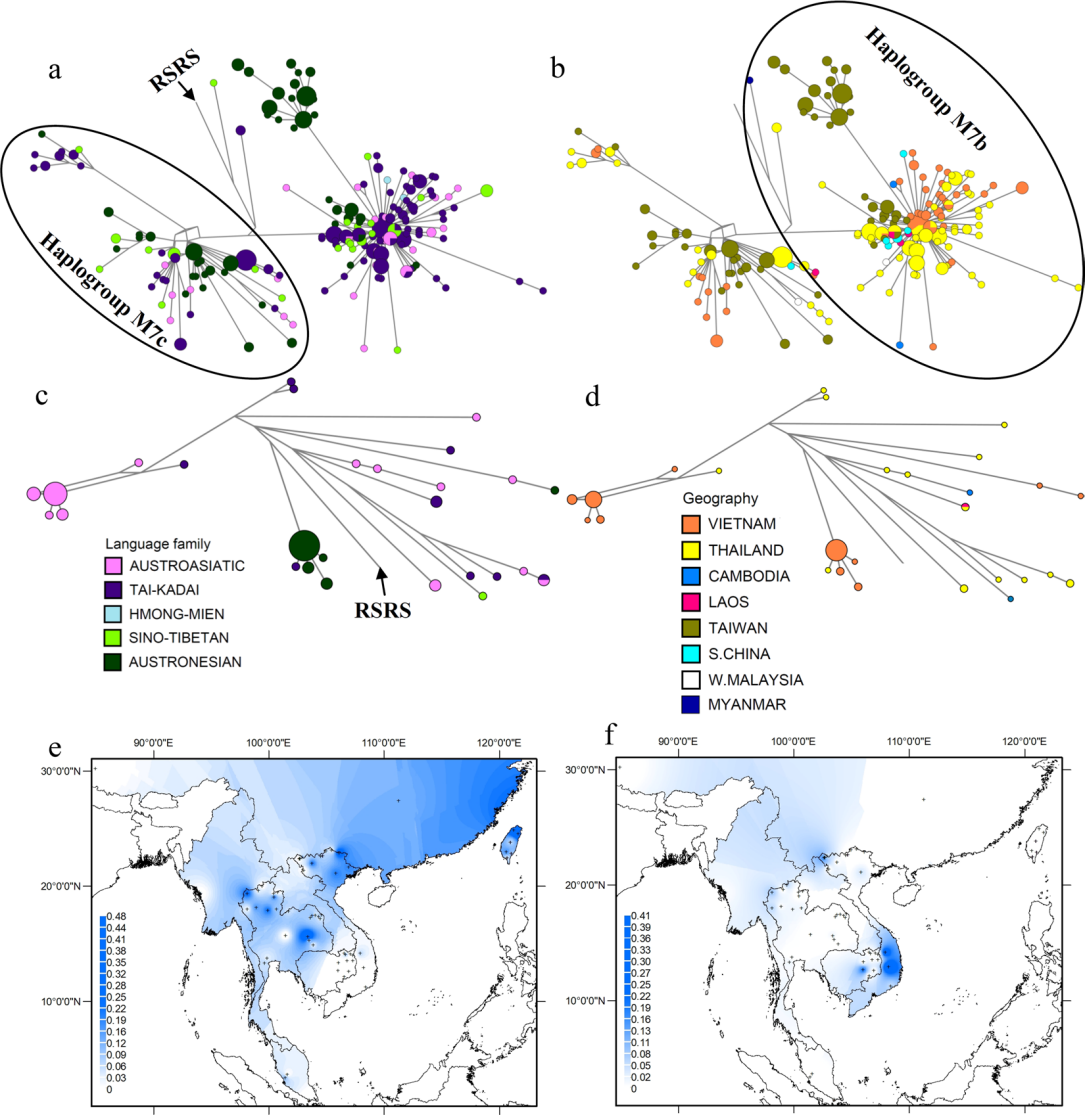
Diversity and distribution of major subhaplogroups of haplogroup M found in MSEA. (**a**,**b**) Network of haplogroup M7 complete mtDNA sequences, color-coded by linguistic affiliation and geographic origin, respectively. (**c**,**d**) Network of haplogroup M71 complete mtDNA sequences, color-coded by linguistic affiliation and geographic origin, respectively. (**e**,**f**) Spatial frequency distribution maps of haplogroups M7 and M71 in MSEA, respectively: the more intense the color, the higher the frequency in the population. Crosses mark the sampling sites of the 68 MSEA populations included in the analysis (see Table S1 in Online Resource 1).

Haplogroup M7 is found in all countries included in this study and here has a coalescence age of ~47 kya (Table S6 in Online Resource 1). It is represented by subhaplogroups M7b and M7c (Table S4 in Online Resource 1; Figure S15 in Online Resource 2) with coalescence times of ~32.6 kya and ~29.4 kya, respectively (Table S6 in Online Resource 1). The network of M7b shows a star-like pattern with numerous sequences shared among language families, indicative of a population expansion (Fig. 7a,b). The central (ancestral) node is found exclusively in TK groups from both Vietnam and Thailand. There is also a cluster of more-distantly related sequences found only in AN groups from Taiwan. Network analysis of haplogroup M7c also shows a signature of an expansion, and overall M7b and M7c are in highest frequency in TK and AN groups (Table S4 in Online Resource 1). Haplogroup M7 reaches the highest frequency in eastern Thailand and northern Taiwan (Fig. 7e).

Haplogroup M71 is found in Thailand, Cambodia, Laos, Myanmar and Vietnam and here has a coalescent age of ~41 kya (Table S6 in Online Resource 1). M71 is one of five major haplogroups in Vietnamese populations. This haplogroup is found in high frequency in AA and AN groups in Vietnam, but is absent from Vietnamese TK, HM, and ST groups (Table S3 in Online Resource 1). However, it does occur in a few TK and ST individuals from outside Vietnam, in addition to some AA individuals (Fig. 7c,d). Subhaplogroup M71 + 151 is restricted in Vietnam to AN groups, but is completely absent from Taiwanese AN groups (Table S1 in Online Resource 1). Overall, haplogroup M71 has the highest frequency in central Vietnam (Fig. 7f).

### Phylogeny of subhaplogroup N9a

**Figure 8:**
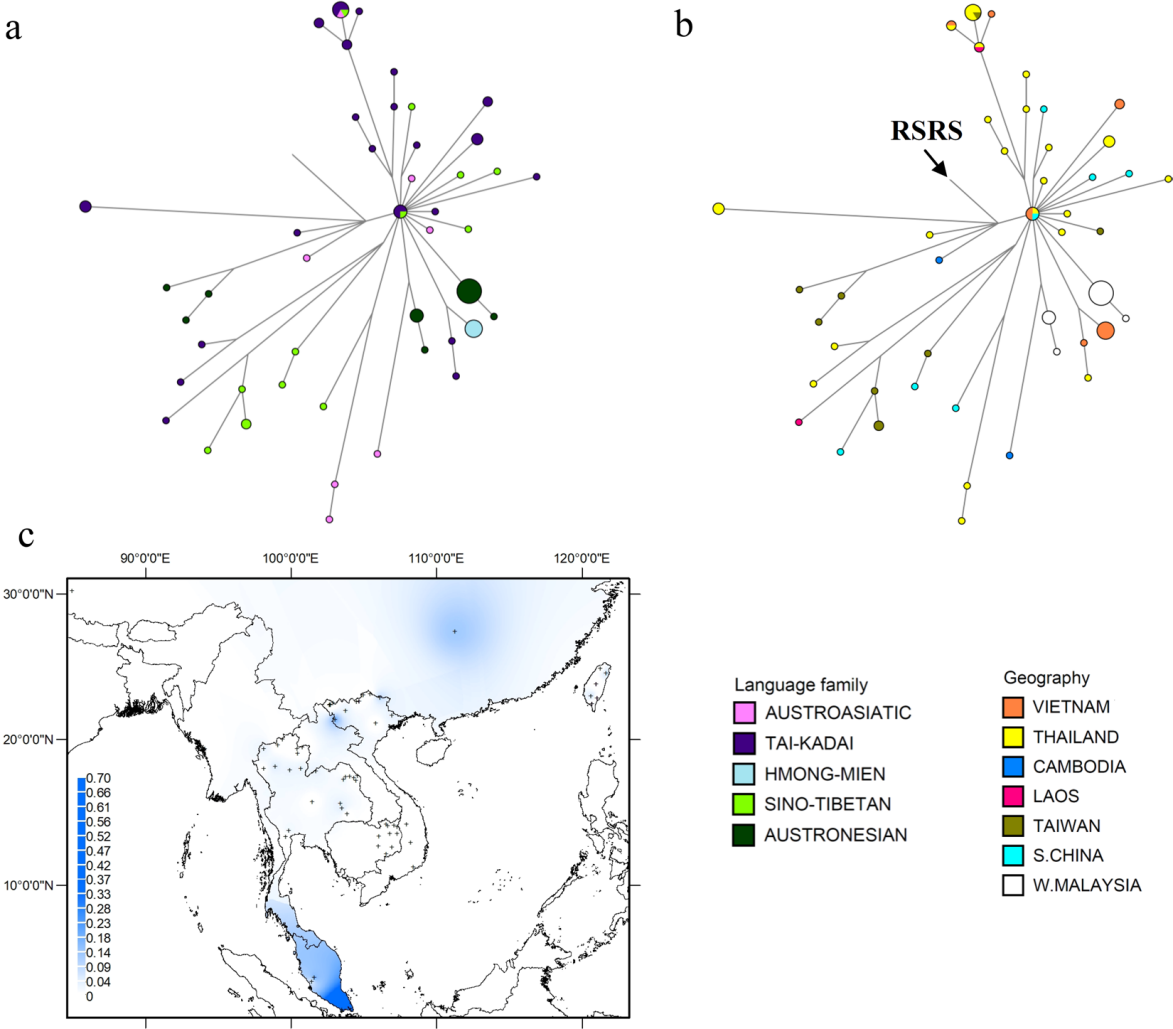
Diversity and distribution of haplogroup N9a. (**a**,**b**) Network of haplogroup N9a complete mtDNA sequences, color-coded by linguistic affiliation and geographic origin, respectively. (**c**) Spatial frequency distribution map of haplogroup N9a in MSEA: the more intense the color, the higher the frequency in the population. Crosses mark the sampling sites of the 68 MSEA populations included in the analysis (see Table S1 in Online Resource 1).

N9a, one of three major sub-clades of haplogroup N, is found in East Asia, Southeast Asia and Central Asia,^1,37,51^ and in MSEA has a coalescence time of ~27 kya (Table S6 in Online Resource 1). There are 14 Vietnamese N9a sequences: 10 fall into subhaplogroup N9a10 + 16311 (branches 2, 3 in Figure S16 in Online Resource 2), one of which (branch 2) shows internal divergence; two into subhaplogroup N9a1, where they represent a new lineage (branch 1; Figure S16 in Online Resource 2); and two into subhaplogroup N9a6, where they also define a new lineage (branch 4; Figure S16 in Online Resource 2). The network shows that the central node is found in TK and ST groups from Vietnam, Thailand and S. China, indicating a rapid spread (Fig. 8a,b). Also, there is a haplotype shared between AA, TK and ST groups from Thailand and Taiwan (Fig. 8a,b). Haplogroup N9a reaches the highest frequency in southern peninsular Malaysia (Fig. 8c), which probably reflects the very high frequency of subhaplogroup N9a6b in the Seletar (Table S1 in Online Resource 1).

## Conclusion

In conclusion, this study adds a large number of complete mtDNA sequences, encompassing all five language families in Vietnam, and refines our understanding of the diversity and distribution of mtDNA haplogroups in MSEA. We identify 111 novel mtDNA lineages, which result in substantially older ages for several haplogroups in MSEA. We also find a peak in the distribution of the differentiation of Vietnamese-specific lineages at around 2.5–3 kya, which corresponds with archaeological evidence for the agriculturally-driven expansion of the Dong Son culture^34^. AN groups from Vietnam have distinct mtDNA haplotypes compared both to other Vietnamese groups, and to AN groups from Taiwan. Finally, network analyses of the major MSEA haplogroups provide evidence of population expansions, with numerous instances of identical or closely-related sequences shared between groups from different language families and/or different geographic regions. Overall, the results of this study highlight the complexity of the mtDNA landscape in MSEA, and point to the need for further studies of the genetic prehistory of this region.

## Electronic supplementary material

Dataset 1

Dataset 2
