# Supplementary material for: Complete human mtDNA genome sequences from Vietnam and the phylogeography of Mainland Southeast Asia
Source: Sci Rep. 2018 Aug 3;8:11651. doi: 10.1038/s41598-018-29989-0 (PMC6076260; doi:10.1038/s41598-018-29989-0)
Supplement: Supplementary file 2 — Dataset 2 [file 41598_2018_29989_MOESM2_ESM.pdf]

Complete human mtDNA genome sequences from Vietnam and the phylogeography of Mainland Southeast Asia

Nguyen Thuy Duong[1#], Enrico Macholdt[2#], Nguyen Dang Ton[1], Leonardo Arias[2], Roland Schröder[2], Nguyen Van Phong[1], Vo Thi Bich Thuy[1], Nguyen Hai Ha[1], Huynh Thi Thu Hue[1], Nguyen Thi Xuan[1], Kim Thi Phuong Oanh[1], Le Thi Thu Hien[1], Nguyen Huy Hoang[1], Brigitte Pakendorf[3], Mark Stoneking[2]* and Nong Van Hai[1]*

[1]Institute of Genome Research, Vietnam Academy of Science and Technology, 18 Hoang Quoc Viet, Cau Giay, Hanoi, Vietnam

[2]Department of Evolutionary Genetics, Max Planck Institute for Evolutionary Anthropology, Deutscher Platz 6, D04103 Leipzig, Germany

[3]Dynamique du Langage, UMR5596, CNRS & Université de Lyon, 69363 Lyon Cedex 07, France

*** Corresponding authors**

1. Associate Professor Nong Van Hai, PhD

Institute of Genome Research, Vietnam Academy of Science and Technology, 18 Hoang Quoc Viet, Cau Giay, Hanoi, Vietnam

Tel: +84 24 3756 2934; Email: vhnong@igr.ac.vn

2. Professor Mark Stoneking, PhD

Department of Evolutionary Genetics, Max Planck Institute for Evolutionary Anthropology, Deutscher Platz 6, D04103 Leipzig, Germany

Tel: +49 341 3550 502; Email: stoneking@eva.mpg.de

# Contributed equally

Supplementary Information

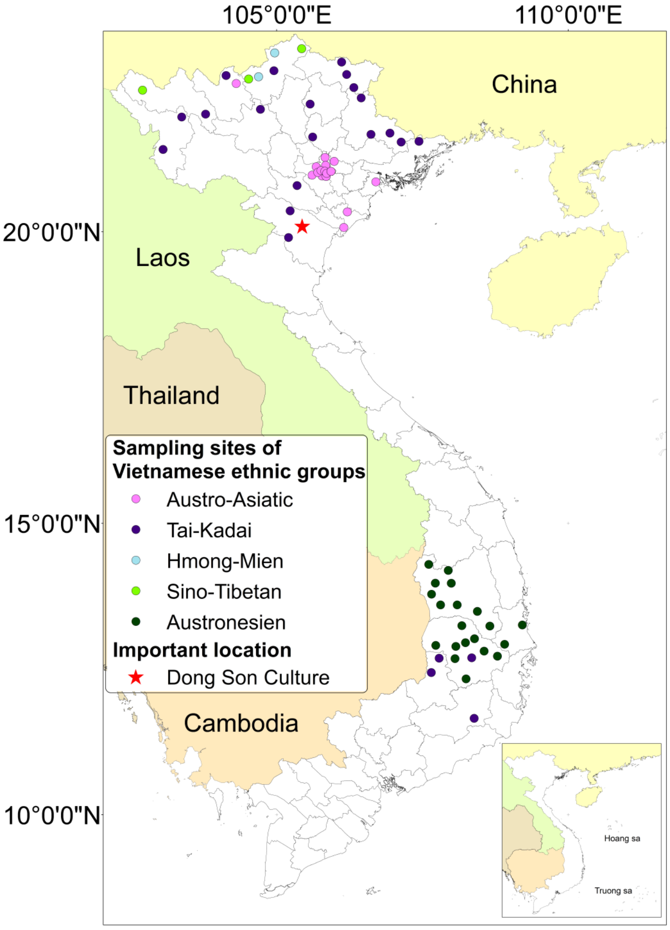

Figure S1. Map showing the sampling sites of the studied populations and their language family affiliation.

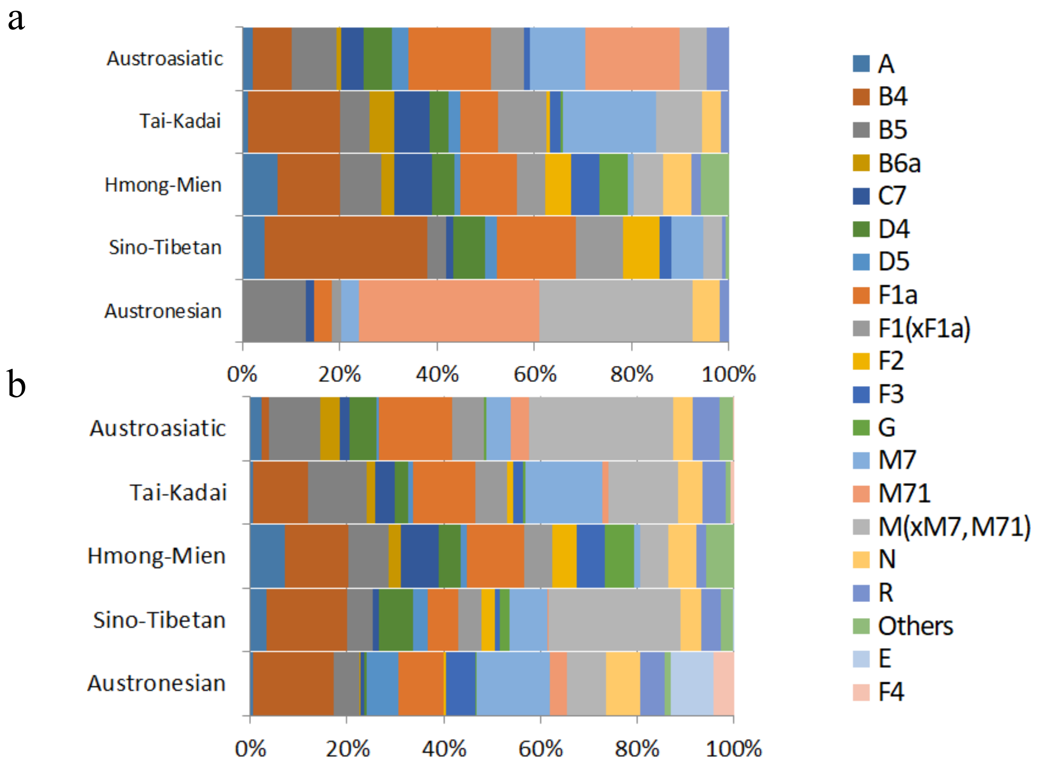

Figure S2. Bar plots showing the relative frequency of major haplogroups across five language families in a) Vietnamese populations; b) MSEA populations (including the current Vietnamese data).

a

b

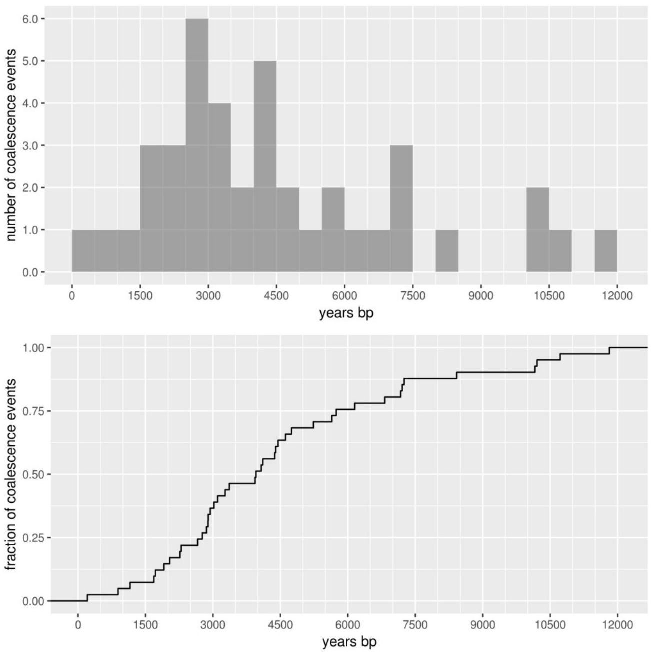

Figure S3. Histogram of coalescence times of Vietnam-specific lineages. a) The number of coalescence events in relation to time; b) The cumulative histogram of the fraction of coalescence events. Time 0 is the present and the x-axis is measured in time before present scaled by the number of coalescence events.

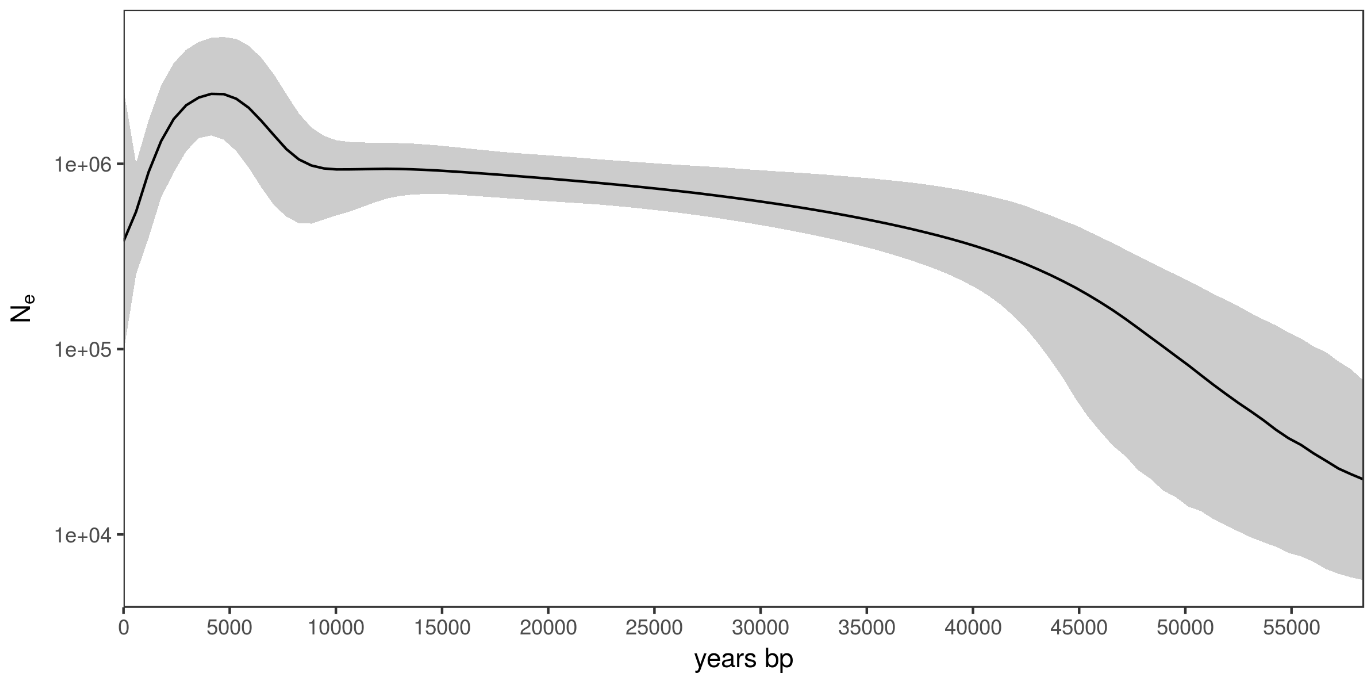

Figure S4. Baysian skyline plot of 609 Vietnamese mtDNA sequences. The thick line indicates the median estimate, bracketed by thin lines for the 95% highest prosterior density.

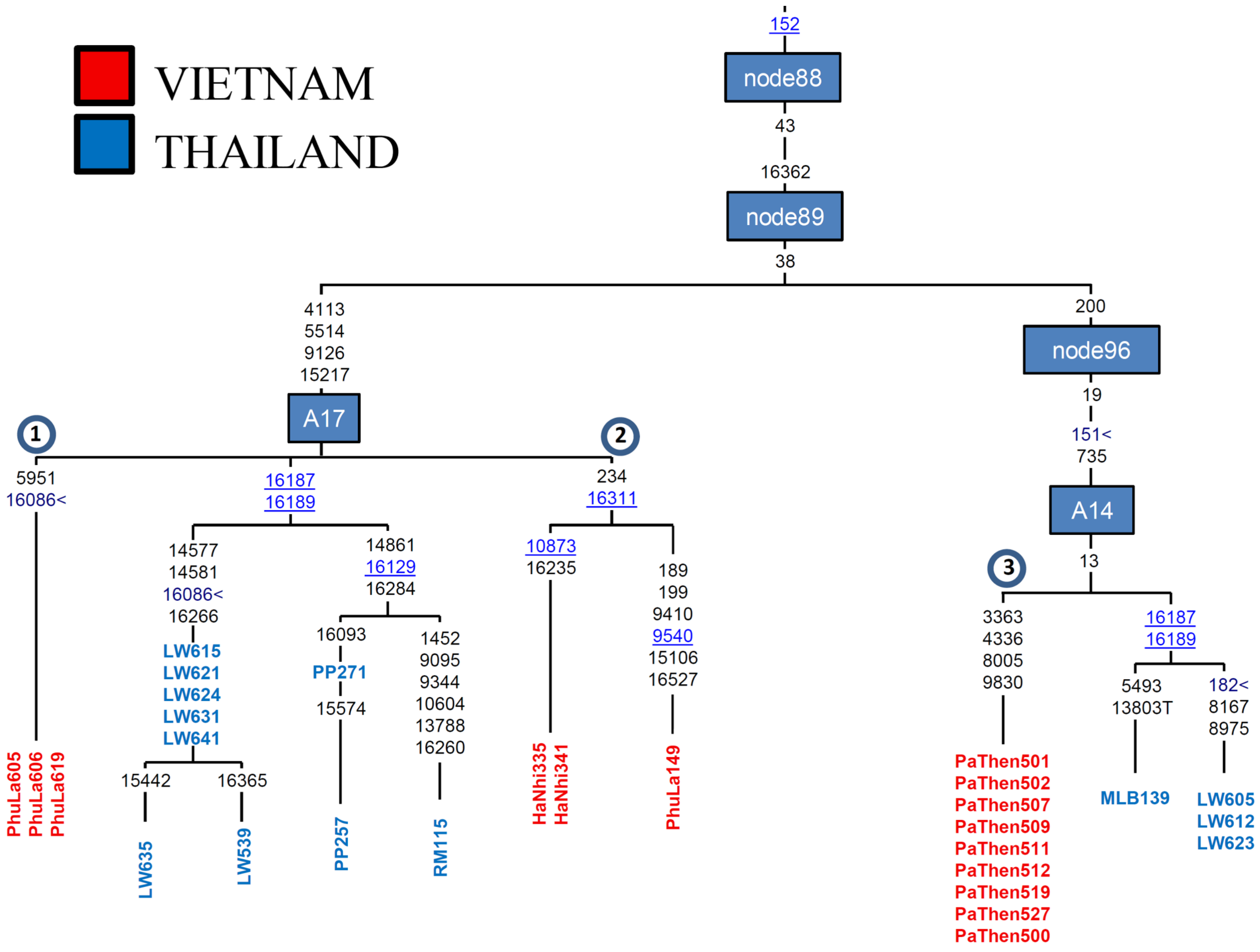

Figure S5. Phylogenetic tree of haplogroups A14 and A17, including MSEA sequences located in the same branches as Vietnamese sequences. Sequence label colors reflect geographic origin as indicated. Circled numbers indicate branches that were either used to estimate the coalescence time of Vietnamese-specific lineages (Table S5), or are referred to in the text.

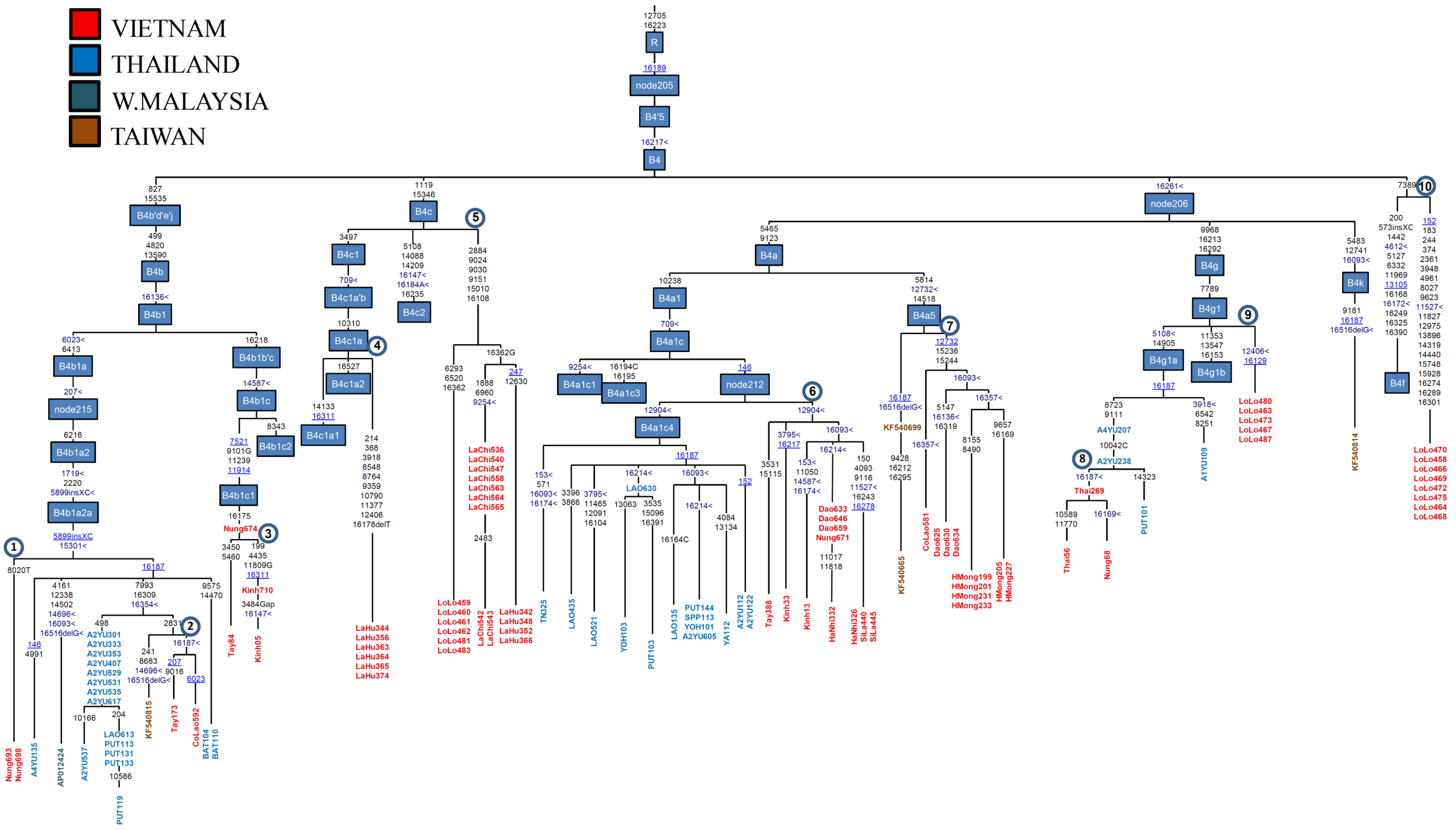

Figure S6. Phylogenetic tree of haplogroup B4, including MSEA sequences located in the same branches as Vietnamese sequences. Sequence label colors reflect geographic origin as indicated. Circled numbers indicate branches that were either used to estimate the coalescence time of Vietnamese-specific lineages (Table S5), or are referred to in the text.

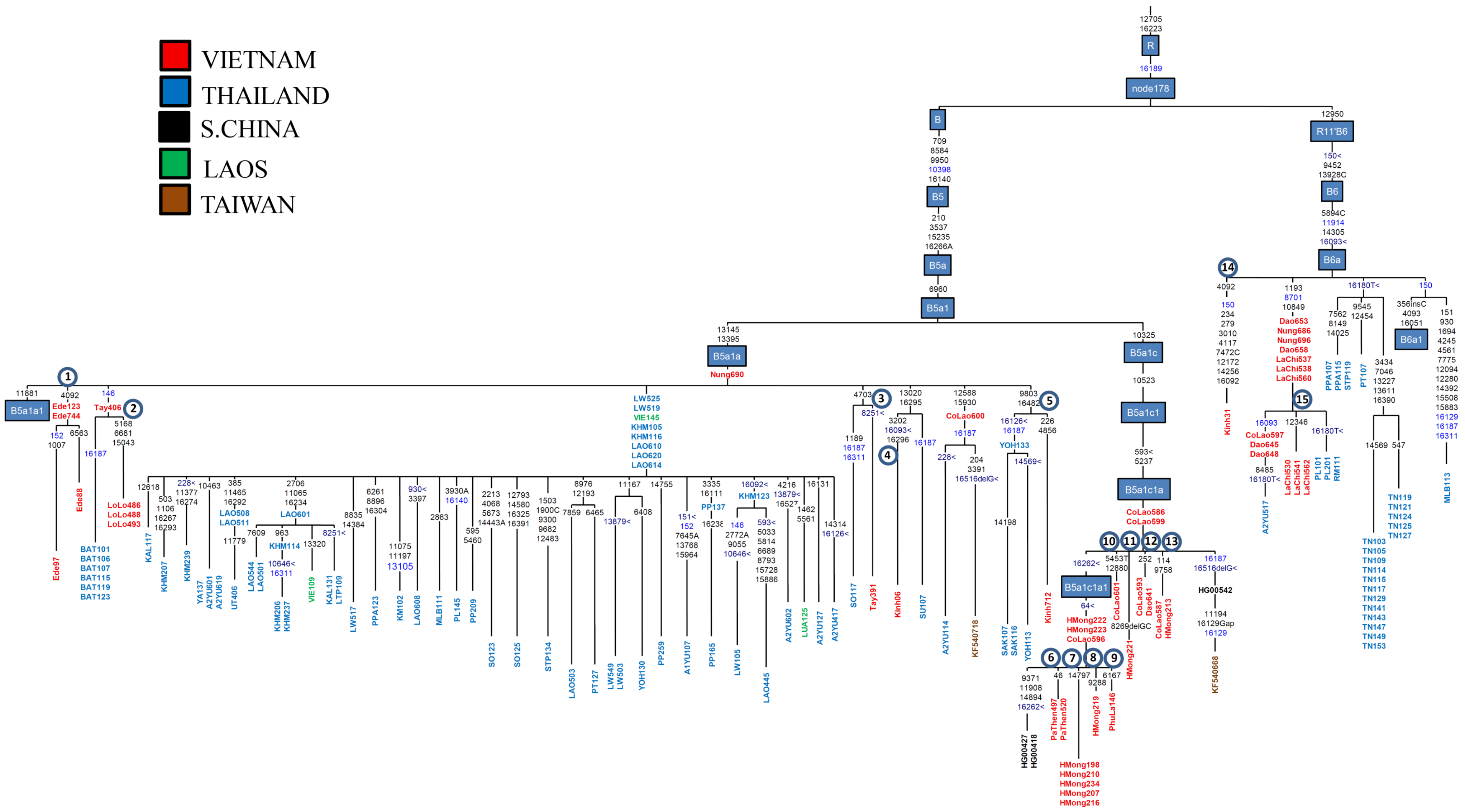

Figure S7. Phylogenetic tree of haplogroups B5 and B6, including MSEA sequences located in the same branches as Vietnamese sequences. Sequence label colors reflect geographic origin as indicated. Circled numbers indicate branches that were either used to estimate the coalescence time of Vietnamese -specific lineages (Table S5), or are referred to in the text.

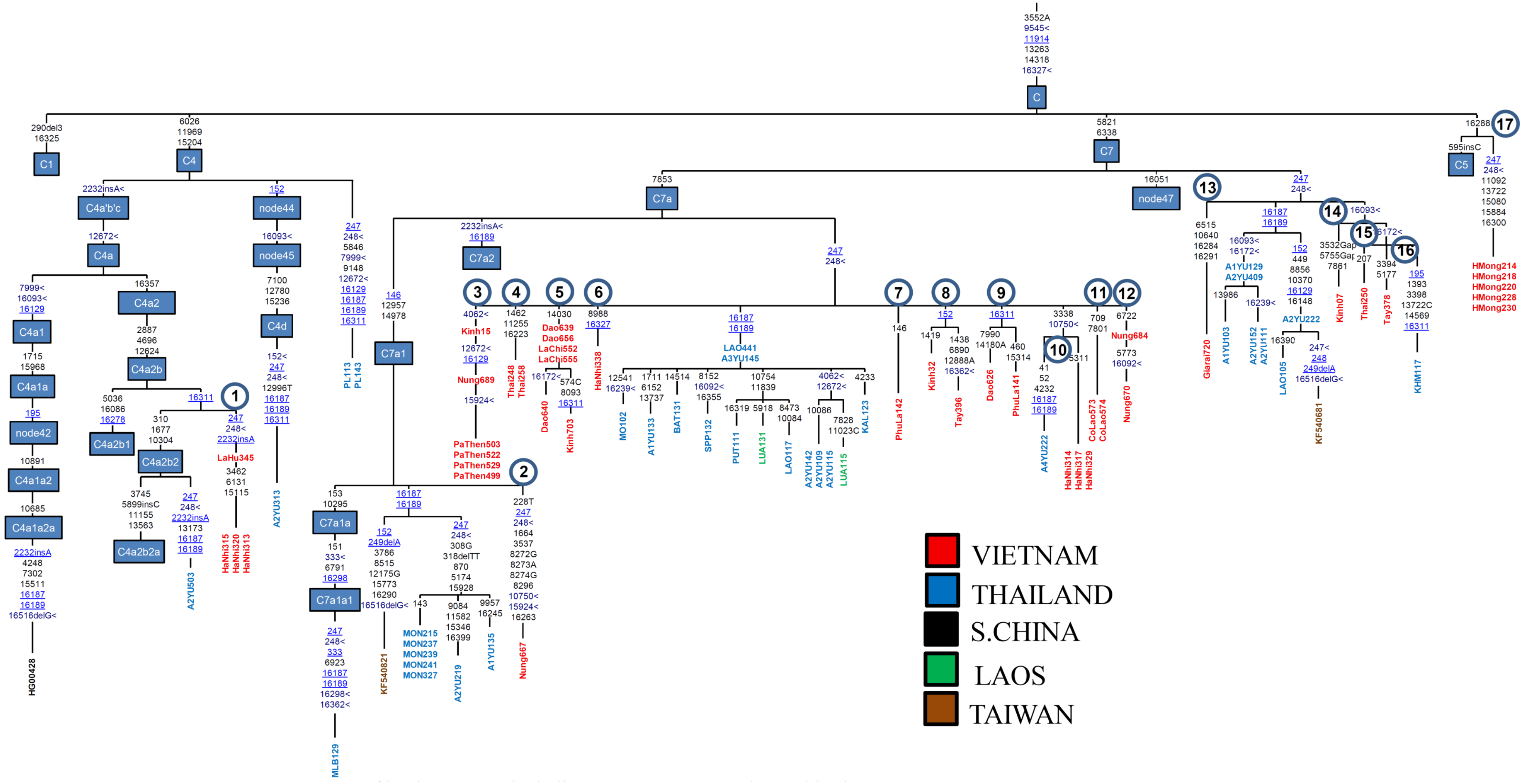

Figure S8. Phylogenetic tree of haplogroup C, including MSEA sequences located in the same branches as Vietnamese sequences. Sequence label colors reflect geographic origin as indicated. Circled numbers indicate branches that were either used to estimate the coalescence time of Vietnamese-specific lineages (Table S5), or are referred to in the text.

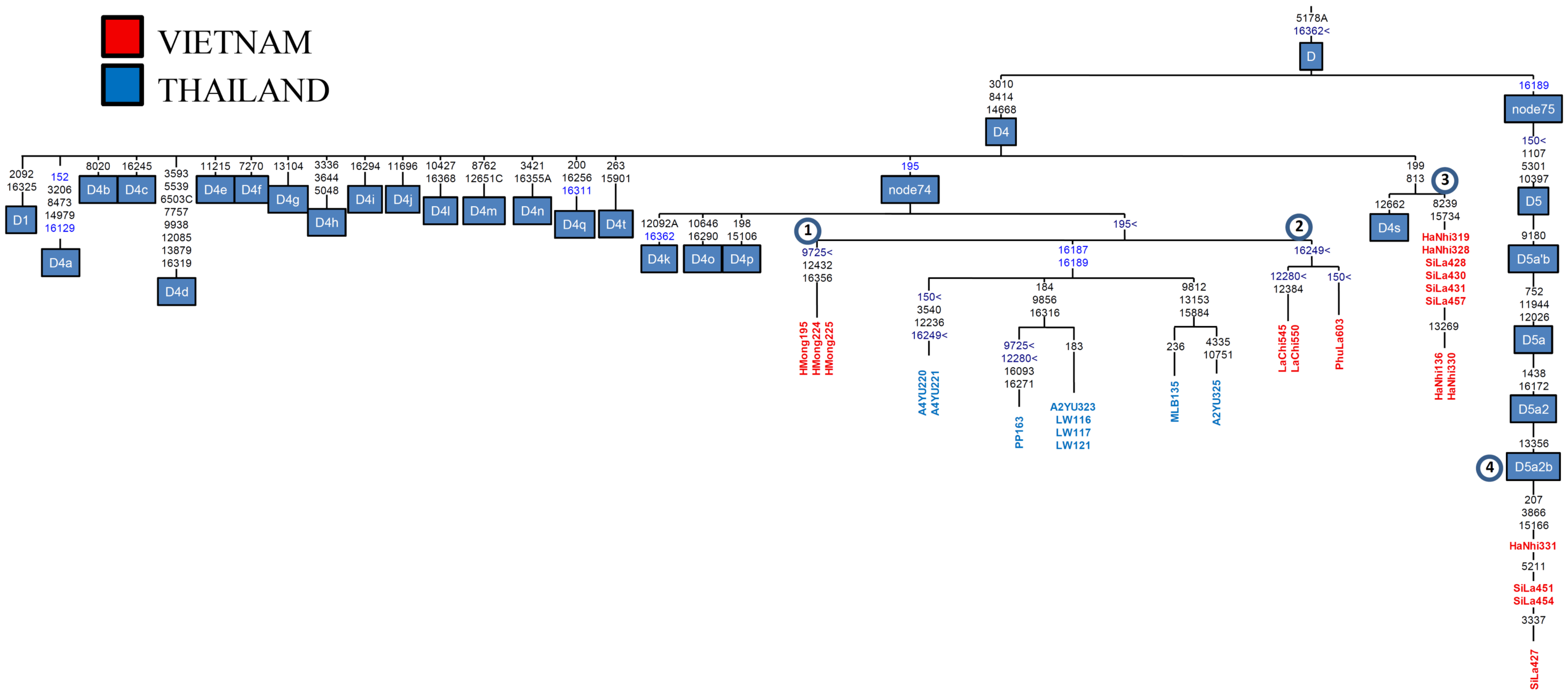

Figure S9. Phylogenetic tree of haplogroup D, including MSEA sequences located in the same branches as Vietnamese sequences. Sequence label colors reflect geographic origin as indicated. Circled numbers indicate branches that were either used to estimate the coalescence time of Vietnamese-specific lineages (Table S5), or are referred to in the text.

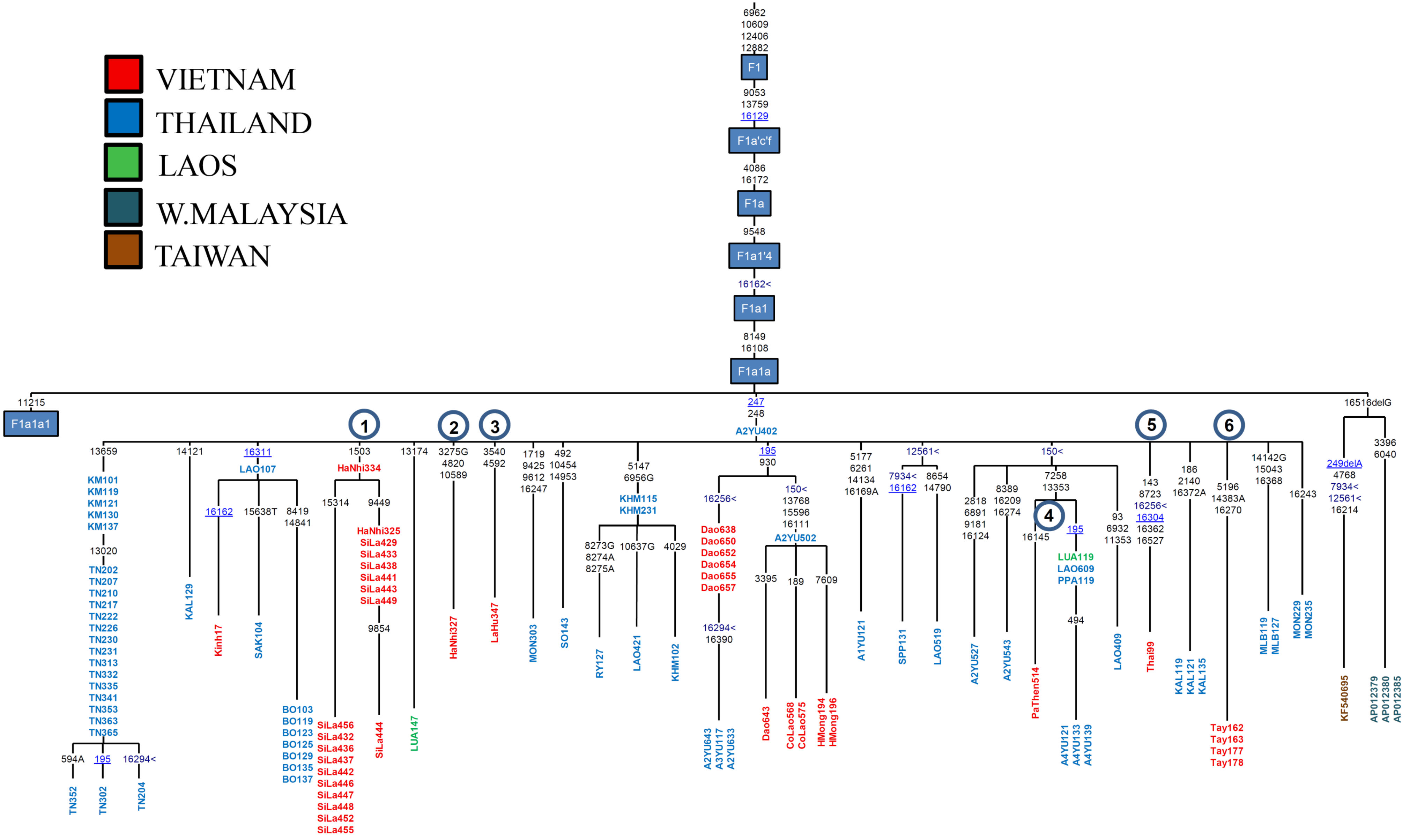

Figure S10. Phylogenetic tree of haplogroup F1a1a, including MSEA sequences located in the same branches as Vietnamese sequences. Sequence label colors reflect geographic origin as indicated. Circled numbers indicate branches that were either used to estimate the coalescence time of Vietnamese-specific lineages (Table S5), or are referred to in the text.

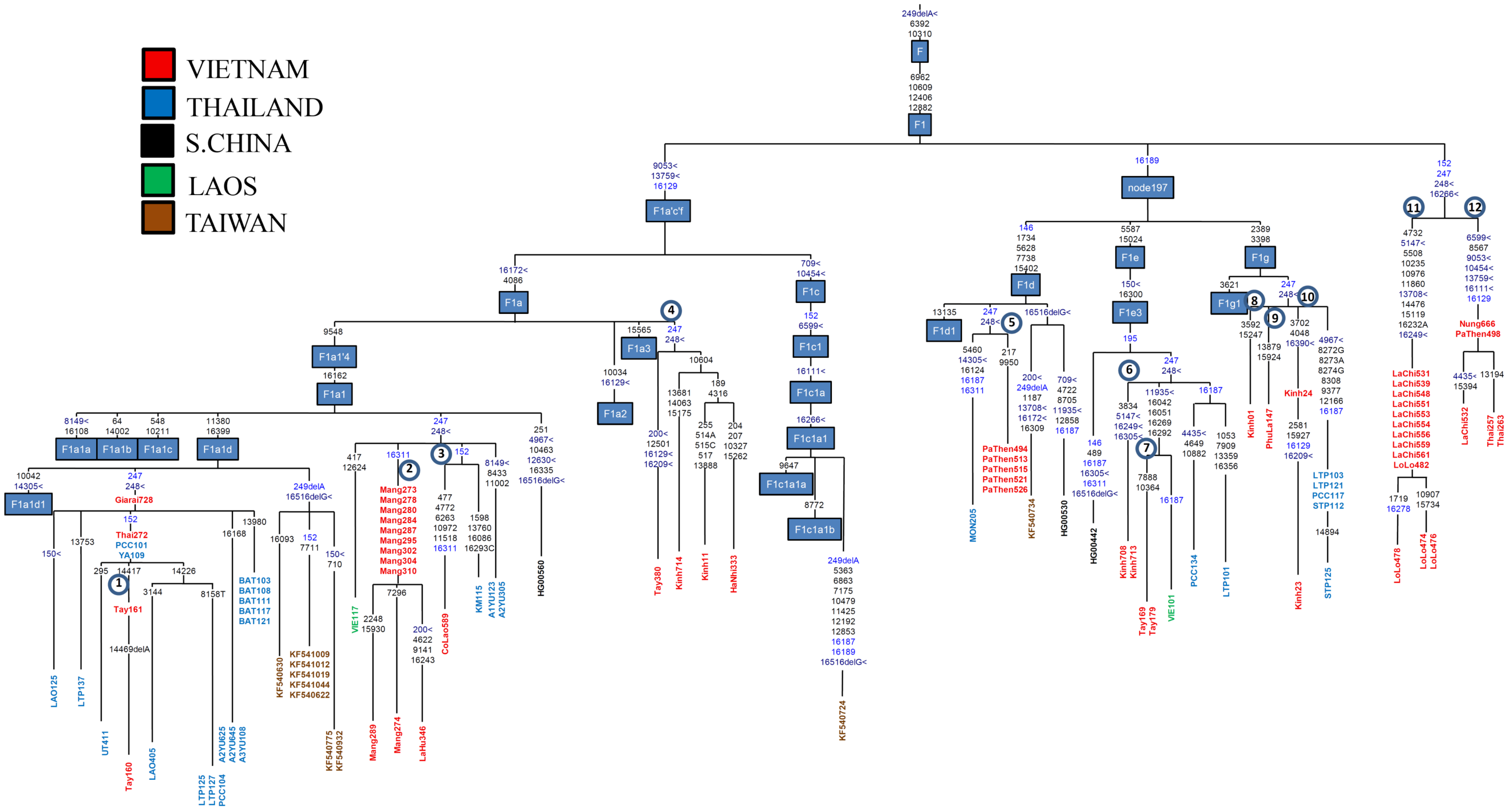

Figure S11. Phylogenetic tree of haplogroup F1 (excluding F1a1a, which is shown in Figure S10), including MSEA sequences located in the same branches as Vietnamese sequences. Sequence label colors reflect geographic origin as indicated. Circled numbers indicate branches that were either used to estimate the coalescence time of Vietnamese-specific lineages (Table S5), or are referred to in the text.

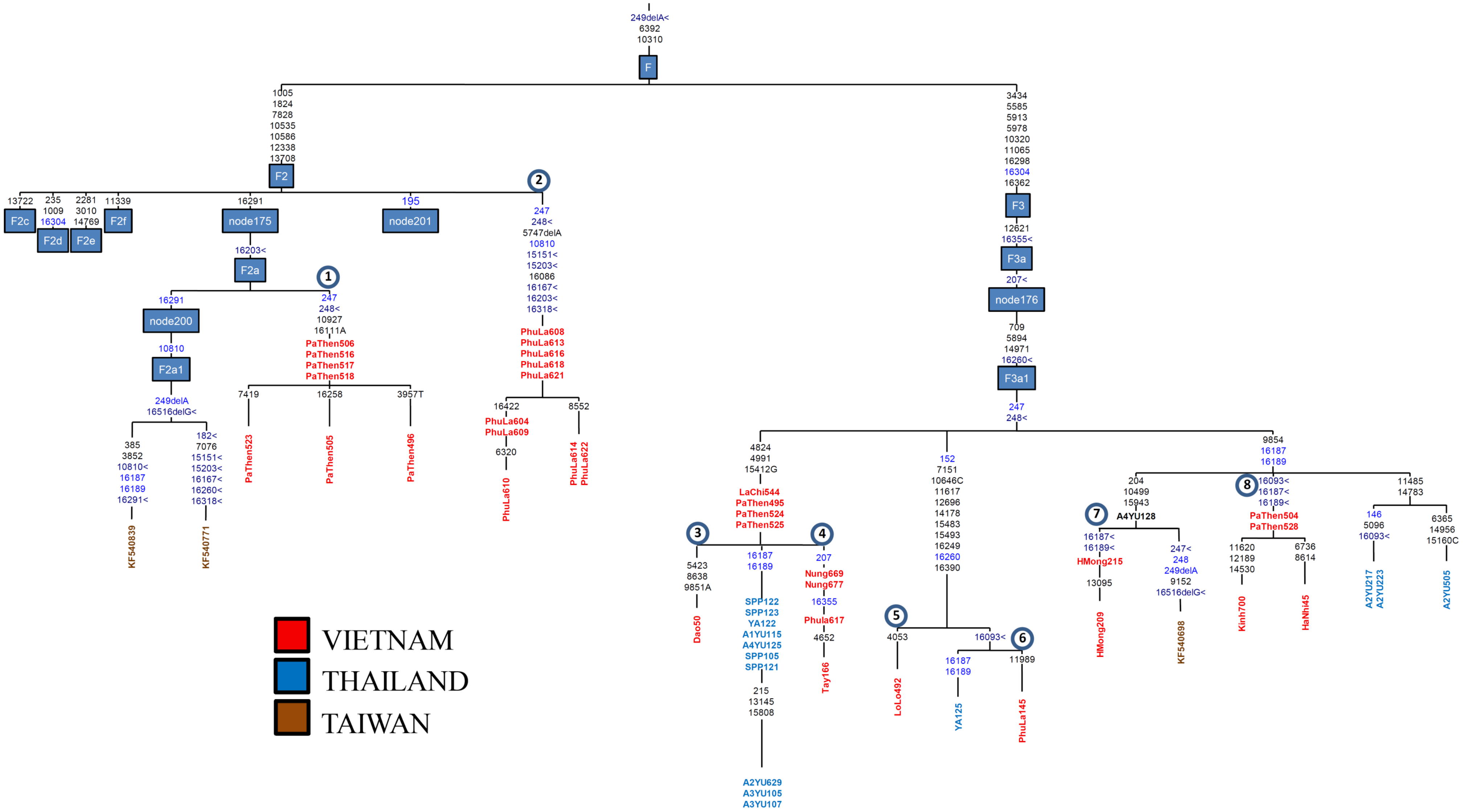

Figure S12. Phylogenetic tree of haplogroups F2 and F3, including MSEA sequences located in the same branches as Vietnamese sequences. Sequence label colors reflect geographic origin as indicated. Circled numbers indicate branches that were either used to estimate the coalescence time of Vietnamese-specific lineages (Table S5), or are referred to in the text.

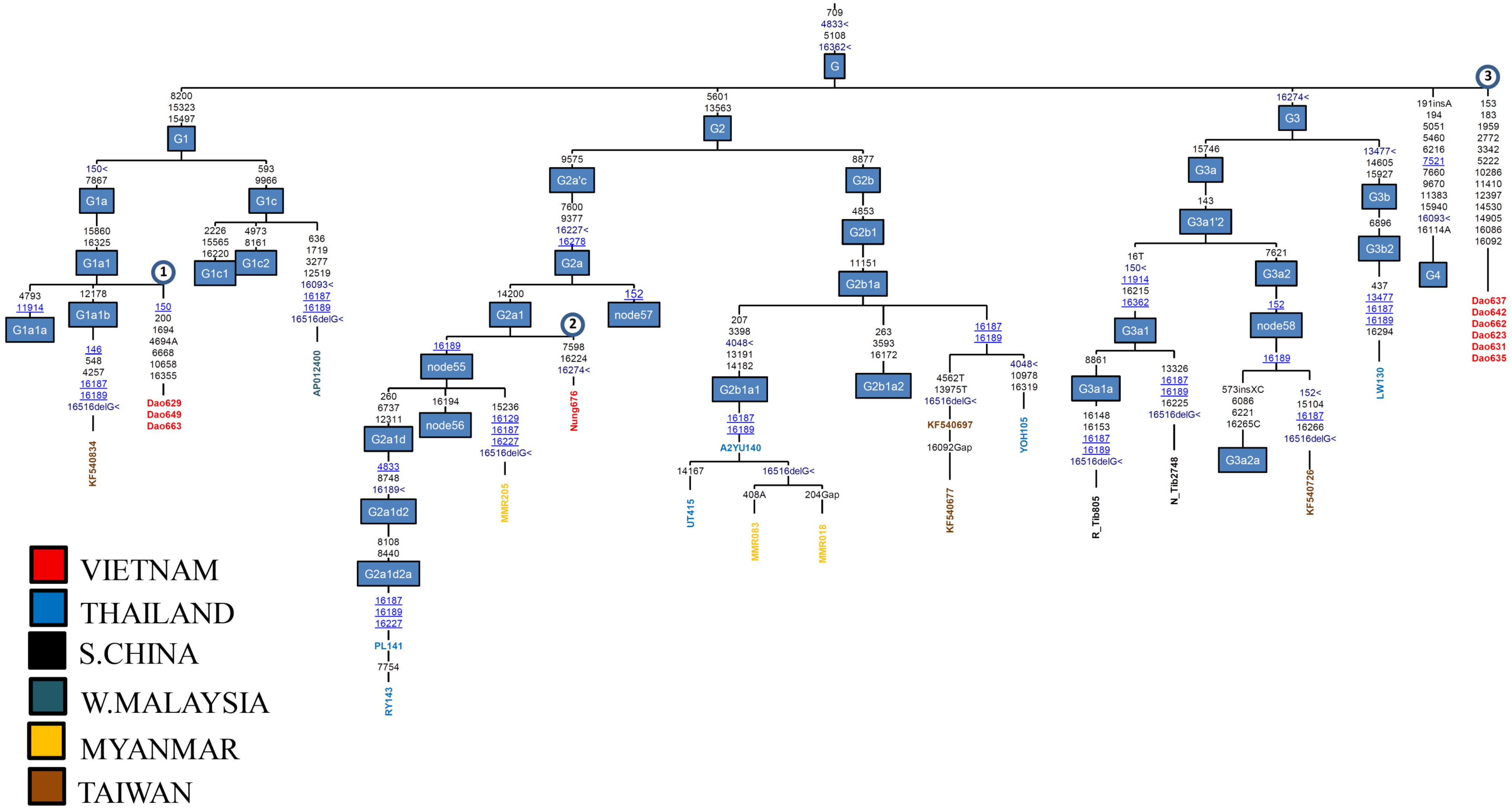

Figure S13. Phylogenetic tree of haplogroup G, including MSEA sequences located in the same branches as Vietnamese sequences. Sequence label colors reflect geographic origin as indicated. Circled numbers indicate branches that were either used to estimate the coalescence time of Vietnamese-specific lineages (Table S5), or are referred to in the text.

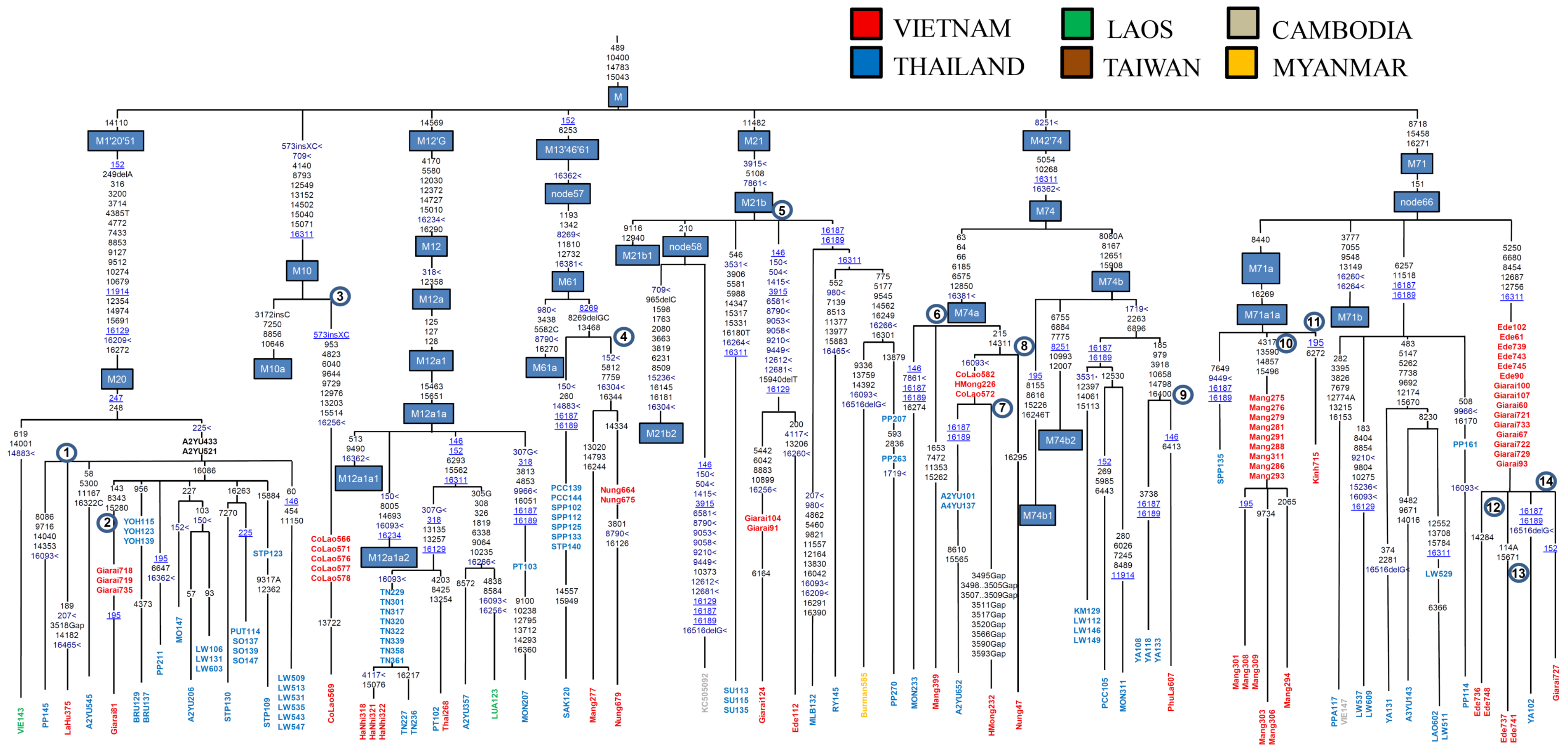

Figure S14. Phylogenetic tree of haplogroup M (excluding M7, which is shown in Figure S15), including MSEA sequences located in the same branches as Vietnamese sequences. Sequence label colors reflect geographic origin as indicated. Circled numbers indicate branches that were either used to estimate the coalescence time of Vietnamese-specific lineages (Table S5), or are referred to in the text.

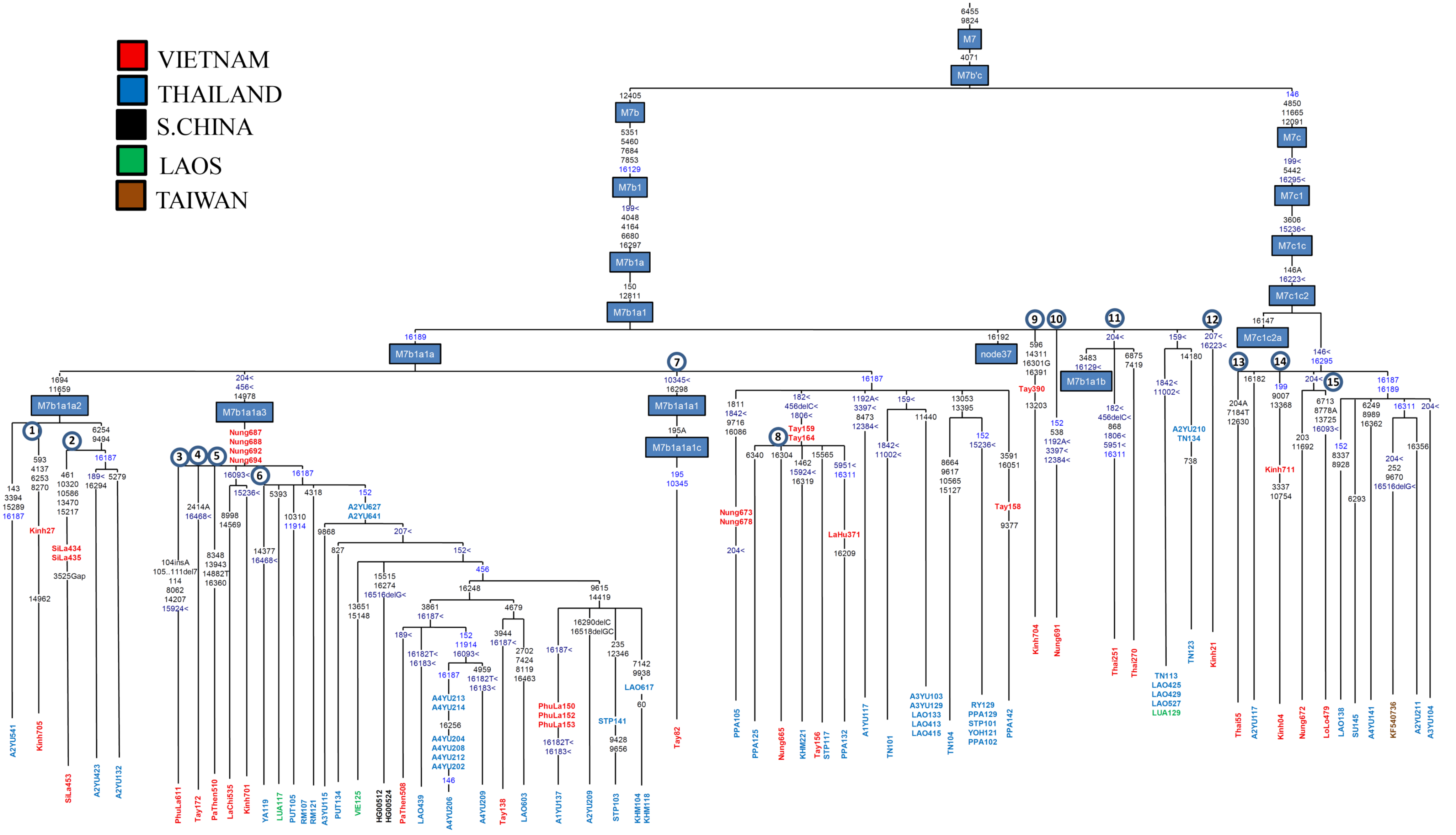

Figure S15. Phylogenetic tree of haplogroup M7, including MSEA sequences located in the same branches as Vietnamese sequences. Sequence label colors reflect geographic origin as indicated. Circled numbers indicate branches that were either used to estimate the coalescence time of Vietnamese-specific lineages (Table S5), or are referred to in the text.

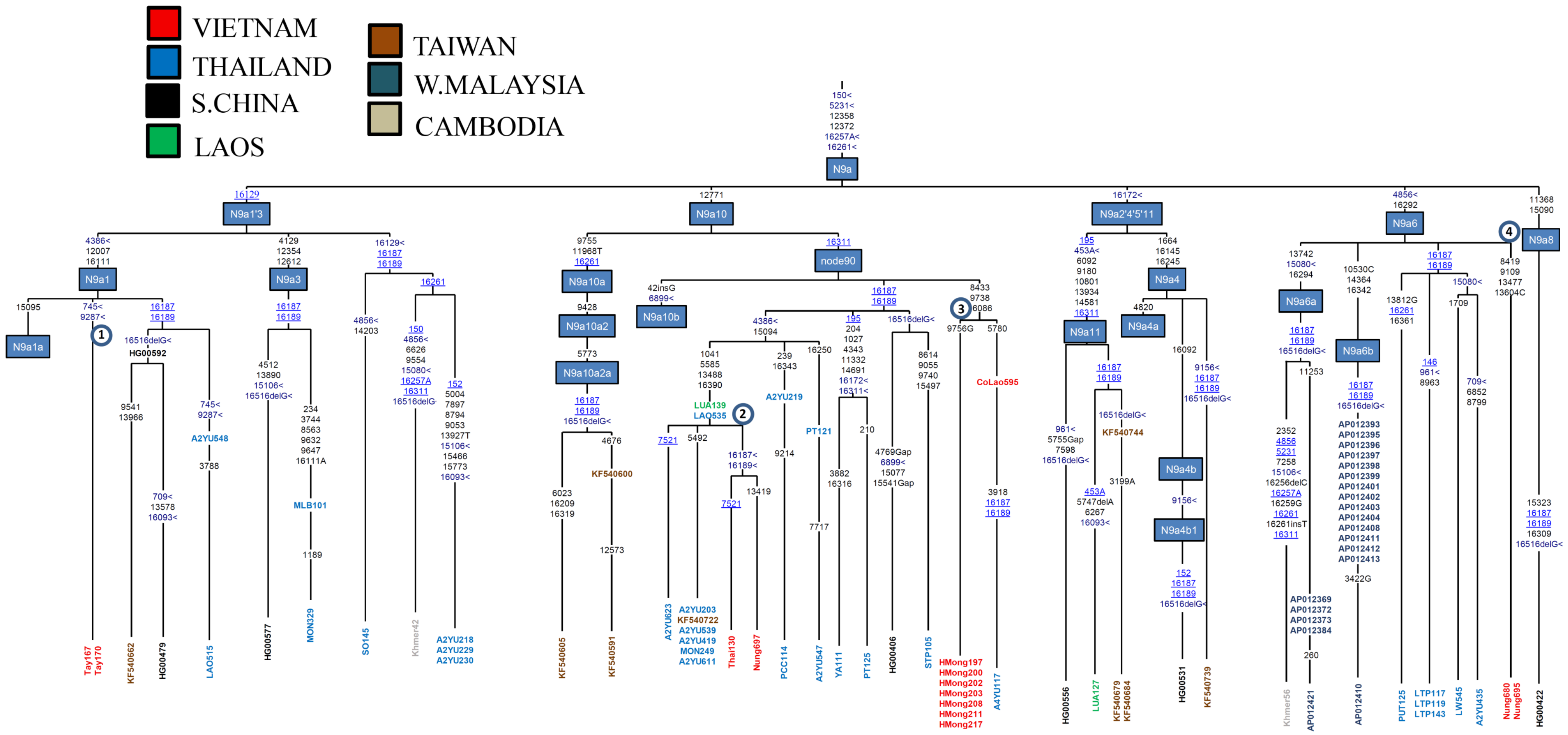

Figure S16. Phylogenetic tree of haplogroup N9a , including MSEA sequences located in the same branches as Vietnamese sequences. Sequence label colors reflect geographic origin as indicated. Circled numbers indicate branches that were either used to estimate the coalescence time of Vietnamese-specific lineages (Table S5), or are referred to in the text.
